# The Musashi 1 Controls the Splicing of Photoreceptor-Specific Exons in the Vertebrate Retina

**DOI:** 10.1371/journal.pgen.1006256

**Published:** 2016-08-19

**Authors:** Daniel Murphy, Benjamin Cieply, Russ Carstens, Visvanathan Ramamurthy, Peter Stoilov

**Affiliations:** 1 Department of Biochemistry, Robert C. Byrd Health Sciences Center, West Virginia University, Morgantown, West Virginia, United States of America; 2 Department of Medicine, University of Pennsylvania, Perelman School of Medicine, Philadelphia, Pennsylvania, United States of America; 3 Departments of Biochemistry, Ophthalmology and Center for Neuroscience, Robert C. Byrd Health Sciences Center, West Virginia University, Morgantown, West Virginia, United States of America; 4 Department of Biochemistry and Cancer Institute, Robert C. Byrd Health Sciences Center, West Virginia University, Morgantown, West Virginia, United States of America; New York University, UNITED STATES

## Abstract

Alternative pre-mRNA splicing expands the coding capacity of eukaryotic genomes, potentially enabling a limited number of genes to govern the development of complex anatomical structures. Alternative splicing is particularly prevalent in the vertebrate nervous system, where it is required for neuronal development and function. Here, we show that photoreceptor cells, a type of sensory neuron, express a characteristic splicing program that affects a broad set of transcripts and is initiated prior to the development of the light sensing outer segments. Surprisingly, photoreceptors lack prototypical neuronal splicing factors and their splicing profile is driven to a significant degree by the Musashi 1 (MSI1) protein. A striking feature of the photoreceptor splicing program are exons that display a "switch-like" pattern of high inclusion levels in photoreceptors and near complete exclusion outside of the retina. Several ubiquitously expressed genes that are involved in the biogenesis and function of primary cilia produce highly photoreceptor specific isoforms through use of such “switch-like” exons. Our results suggest a potential role for alternative splicing in the development of photoreceptors and the conversion of their primary cilia to the light sensing outer segments.

## Author Summary

Vertebrates possess extraordinarily complex nervous systems that are formed by hundreds of different types of neurons. This neuronal diversity is achieved in part through the expression of specific sets of genes. In addition, neurons use alternative splicing to a much higher degree than other cells to produce neuronal variants of broadly expressed proteins. Here we characterize the transcriptome of a single neuronal type, the vertebrate photoreceptors. We find that photoreceptors similar to other neurons utilize large number of alternative exons. Surprisingly, the splicing profile of photoreceptor cells and the factors that control it diverge from those of central nervous system and inner retinal neurons. We find multiple examples of genes producing photoreceptor specific splicing variants that completely replace the broadly expressed protein isoforms. Several of these genes participate in the formation of the primary cilium and the elaborated outer segments that are needed for light perception. Our work implies that alternative splicing may enable photoreceptors to acquire their characteristic shape and perform their light sensing function.

## Introduction

Vertebrate nervous systems contain numerous types of neurons with characteristic morphology, connectivity, electrophysiological properties, and neurotransmitter signatures. Single cell transcriptome profiling studies reveal dozens of distinct gene expression profiles in the central nervous system (CNS) and the retina, suggesting that neuronal cell identity is established and maintained by specific gene expression programs [1–5]. A limitation of the single cell approaches is the relatively low coverage of the transcriptome that is biased towards the 3'-end of the transcripts [6]. The depth and distribution of the reads produced by the current single cell transcriptome profiling approaches do not allow the reliable assessment of the levels of transcript isoforms produced by alternative splicing. Thus, the posttranscriptional layer in the regulation of gene expression in neurons, which is required for the normal development and function of the CNS, remains hidden [7–12].

Alternative pre-mRNA splicing is a major mechanism for generating protein diversity in vertebrates. In particular, neurons use alternative splicing for generating protein diversity to a significantly higher degree than any other cell type [13,14]. Characteristically, neurons broadly utilize microexons that are defined by different groups as being no longer than 27nt or 51nt [10,15]. The neuronal splicing program and the inclusion of neuronal microexons are governed by splicing factors belonging to several families of RNA binding proteins: PTBP, ELAVL, NOVA, KHDRBS, SRRM and RBFOX [14,16–19]. With the exception of PTBP1, these proteins have high expression levels in neurons and are not expressed or have limited expression outside of the nervous system. PTBP1, which represses splicing of neuronal exons outside of the nervous system, is replaced by the PTBP2 in the early stages of neuronal differentiation.

Retinal photoreceptor cells provide an intriguing model to study how gene expression programs shape the cell structure and properties. Photoreceptors have a distinct morphology with a characteristic light sensing organelle termed the outer segment. The photoreceptor outer segment is a sensory cilium with an elaborate structure of membrane stacks. Surprisingly, the genes involved in the biogenesis and maintenance of the photoreceptor cilium are ubiquitously expressed in all ciliated cells. Recently, isoforms for two of these proteins, Arl6 (BBS3) and Ttc8 (BBS8), were shown to be preferentially expressed in photoreceptors [20–23]. The retinal variant of Arl6, a Ras related GTP-binding protein, is required for the survival of zebrafish photoreceptor cells and disruption of the gene in mice results in retinal defect [20,21]. These findings raise the possibility that photoreceptor cells are at least in part shaped by post-transcriptional processes such as alternative pre-mRNA splicing.

Here we use animal models to characterize in depth the alternative splicing profiles of photoreceptor cells, a sensory neuron type. We find that photoreceptors express a characteristic splicing program that includes a set of highly photoreceptor-specific isoforms. Surprisingly, key neuronal splicing regulators are either not expressed or downregulated in photoreceptor cells. We show that Musashi 1 (MSI1) promotes the splicing of photoreceptor specific exons as part of a combinatorial mechanism that controls splicing in photoreceptor cells.

## Results

### Isolation the photoreceptor transcriptome

**Fig 1:**
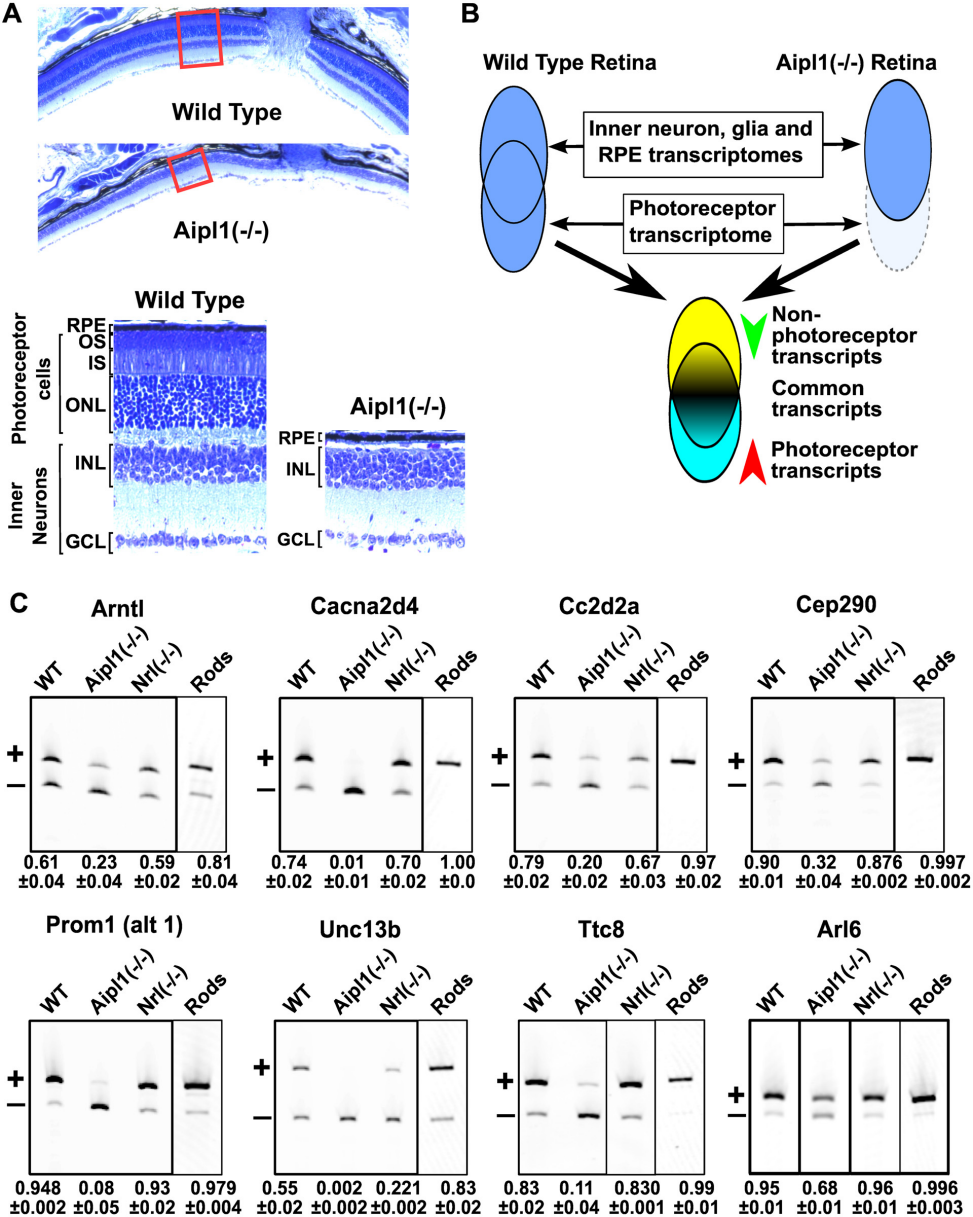
Identification of differentially spliced exons in photoreceptors. **(A)** Retinal sections from wild type and Aipl1(-/-) mice stained with toluidine blue at postnatal day 50. Low magnification images show the overall retinal structure near the site of the optic nerve (top). Red rectangles indicate the position of the magnified images shown below. Below, high magnification images show the layered retinal structure. The Aipl1(-/-) animals lack layers formed by the photoreceptor cells: outer nuclear layer (ONL), inner segment (IS) and outer segment (OS). The retinal pigmented epithelium (RPE), the inner nuclear layer (INL) and ganglion cell layer (GCL) are intact in the Aipl1(-/-) animals. **(B)** Experimental approach for identifying transcripts differentially expressed in photoreceptors. The retina transcriptome is an aggregate of the transcriptomes of multiple cell types. Approximately 40–60% of the cells in the neural retina are photoreceptors. Due to the abundance of photoreceptors in the retina, their loss produces changes in the retinal transcriptome that are readily detectable. **(C)** RT-PCR analysis of the inclusion levels of exons identified in the RNA-Seq analysis in retina from wild type, Aipl1(-/-), Nrl(-/-) mice and flow sorted rod photoreceptors (labeled Rods). The exons include the previously described photoreceptor specific exon 2A in the Ttc8 gene and retina enriched exon 6 in the Arl6 gene. The bands corresponding to the exon skipped and exon included mRNA isoforms are labeled with ‘+’ and ‘-’, respectively. The relative exon inclusion and standard error of three independent replicates are shown below each lane.

To identify the features of the retina transcriptome that are specific to photoreceptor cells we analyzed the transcriptomes of retina samples from wild type and Aipl1 knockout mice by RNA-Seq. AIPL1 is a molecular chaperone that is required for photoreceptor survival and by postnatal day 30, the retina of Aipl1(-/-) mice is devoid of photoreceptors (Fig 1A) [24]. Apart from the missing photoreceptors, the Aipl1(-/-) retina has grossly normal anatomy (Fig 1A) [24]. In a comparison between the transcriptomes of Aipl1(-/-) and wild type retina, transcripts with higher expression levels in photoreceptors will appear downregulated in the Aipl1(-/-) sample due to the altered cell composition (Fig 1B). Conversely, transcripts expressed at higher levels in the inner neurons will show elevated expression levels in the Aipl1 knockout retina.

We validated our approach by performing gene level expression analysis and tracking the levels of transcripts specific to photoreceptors. We identified 5377 genes with more than 2 fold difference in their expression level between Aipl1(-/-) and wild type retina (S1 Table). In the Aipl1 knockout, we observed loss of genes known to encode photoreceptor-specific transcription factors, (e.g. NR2E3, NRL), proteins involved in phototransduction, (e.g. RHO, CNGA1, PDE6B), and photoreceptor morphogenesis (e.g. PRPH2, ROM1, FSCN2) (S1 Table). The genes with higher expression in the wild type retina compared to the Aipl1 knockout, showed enrichment of Gene Ontology (GO) categories directly related to photoreceptor development, structure and function (S2A and S2B Table). This enrichment is consistent with the loss of photoreceptor cells in the Aipl1 knockout. The genes with lower expression levels in the wild type retina were part of broad GO categories related to organ development, neuronal cell structure and function (S2A and S2C Table). Thus, the gene level expression data demonstrates that comparing the retinal transcriptome of Aipl1 knockout with that of the wild type retina correctly identifies the transcripts characteristic to photoreceptors.

### Photoreceptors express a characteristic splicing program

We next determined the inclusion levels of alternative exons in the mouse retina (S3 Table). Hierarchical clustering shows that the retinal samples form a separate cluster with a splicing profile in part related to that of other neuronal tissues (S1 Fig). Similar to central nervous system samples, the retina utilizes a significant number of microexons (S1 Fig).

**Fig 2:**
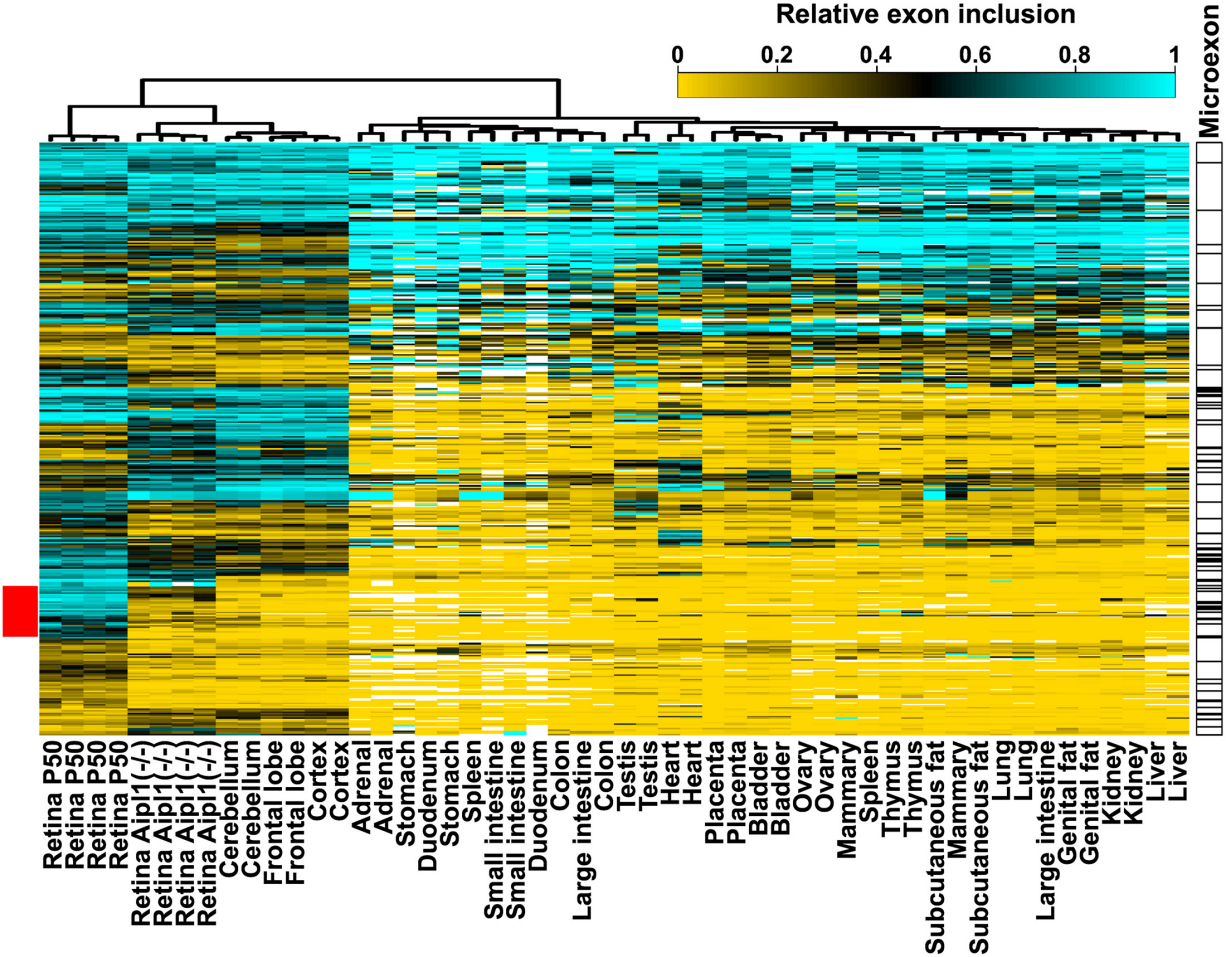
The photoreceptors express a splicing program that is distinct from the splicing profiles of CNS or other retinal neurons. Heatmap showing the relative inclusion levels of exons differentially spliced between the retina of wild type and Aipl(-/-) animals across a panel of mouse tissues. Exon inclusion levels can only be calculated if the transcript is expressed. Thus, exons in transcripts that are not expressed in the majority of the samples are not included in the heatmap. Missing data points are in white color. Unsupervised hierarchical clustering places retinal samples from Aipl1(-/-) mice along with the CNS samples, while the wild type retina samples form a separate cluster. Microexons with length of 30nt or less are annotated on the right. The red bar on the left shows a cluster of photoreceptor specific exons with “switch like” splicing pattern.

We analyzed the differences in exon inclusion levels between the wild type retina the retina of the Aipl1 knockout. Approximately 40% of the differentially spliced exons between wild type and Aipl1 knockout retina were not annotated in the GRCm38 mouse genome assembly. The large number of novel exons prompted us to use Cufflinks to carry out guided transcriptome assembly based on the ENSEMBL GRCm38 annotation and our RNA-Seq data. We then repeated the analysis of the differential splicing using the updated annotation and identified 540 differentially spliced exons in 372 genes (S3 Table). Of these, 318 exons showed higher inclusion levels in wild type retina and 222 had lower inclusion levels. Alternative exons in the Bsg and Ttc8 (Bbs8) genes that are known to be used exclusively in photoreceptors were among the exons with higher inclusion levels in wild type retina, verifying that our approach correctly identifies photoreceptor-specific exons [22,23,25]. We used RT-PCR to test 18 alternative exons that showed differences in inclusion level between 10% and 90% in our RNA-Seq data. All differences in exon inclusion level that were predicted by RNA-Seq were confirmed by the RT-PCR experiment (Fig 1C and S2 Fig). Exons in multiple genes such as Cep290, Cc2d2a, Cacna2d4, Prom1 and Kif1b showed large differences in inclusion levels between the wild type and Aipl1(-/-) retina consistent with a “switch-like” splicing pattern. Similar to the Bsg and Ttc8 exons, these “switch-like” exons appear to be included at high levels in photoreceptors and skipped in all other tissues we examined (Figs 1B and 2).

The photoreceptor splicing pattern we inferred may be due in part to splicing changes in the neurons of Aipl1(-/-) retina in response to the loss of the photoreceptors. To rule out this scenario we analyzed by RT-PCR the inclusion levels of 18 alternative exons in flow sorted rod photoreceptors. The inclusion levels of the majority of the tested exons in rod photoreceptors (17 out of 18) were in concordance with the inferred photoreceptor splicing pattern (Fig 1 and S2 Fig). As expected, the levels of the predicted photoreceptor-specific variants were higher in the isolated photoreceptors compared to the whole retina sample, where the signal is derived from a mixed cell population.

Unsupervised hierarchical clustering of the mouse tissue panel based on the inclusion levels of the exons differentially spliced in photoreceptors places the retina along with the other neuronal tissues (Fig 2). In this clustering the profile of the Aipl1(-/-) retina is more closely related to that of the CNS samples than the wild type retina. The expression of a distinct splicing profile by the photoreceptor cells is likely responsible for the separation of the Aipl1 knockouts from the cluster containing the wild type retinal samples.

As cone photoreceptors comprise only 3% of the retina, it was unclear if the splicing profile we discovered is shared between photoreceptors of different types or if it is specific to rod photoreceptors. To determine if rods and cones share the same splicing program we analyzed the splicing in the retina of Nrl knockout mice by RT-PCR. Disruption of Nrl, a rod-specific transcription factor leads to the conversion of all rod photoreceptors into cone like cells that present the characteristic ultrastructural, histological, molecular and electrophysiological features of cone photoreceptors [26,27]. All tested exons, with exception of an exon in the Glb1l2 (Galactosidase, Beta 1-Like 2) gene, showed identical inclusion levels in the wild type and Nrl knockout retina (Fig 1B and S2 Fig). Thus, rods and cones share largely the same splicing program.

We next carried out gene ontology enrichment analysis to determine if alternative splicing in photoreceptors modifies particular processes or cellular components (S4 Table). Several of the enriched categories point to a significant impact of alternative splicing on the cytoskeleton of photoreceptor cells. Apart from the cytoskeleton we see enrichment of genes in broadly defined categories that are partially related to cell differentiation and neurogenesis, demonstrating that alternative splicing modifies multiple systems and processes in the photoreceptor cells.

### The photoreceptor splicing program is executed prior to photoreceptor morphogenesis

To gain insight into the developmental mechanisms that control splicing in photoreceptors, we analyzed exon inclusion levels in a panel of published retinal RNA-Seq datasets from wild type mice and genetic models that disrupt normal photoreceptor development. In addition to the Aipl1 and Nrl knockouts described above, these models include a Crx knockout [28], a Crx-dominant negative (Crx-DN) mutant [29], and the RD10 mutant [30]. Deletion of Crx or expression of the CRX-DN protein block the transcription of the genes involved in phototransduction and the development of the outer segment [28,29,31]. The RD10 mutant, similar to the Aipl1 knockout, loses its photoreceptors in adulthood [30]. The wild type samples included retina from postnatal day 2, which contain early post-mitotic rod photoreceptor progenitors, and fully developed retina from juvenile and adult animals (postnatal days 21 and 50).

**Fig 3:**
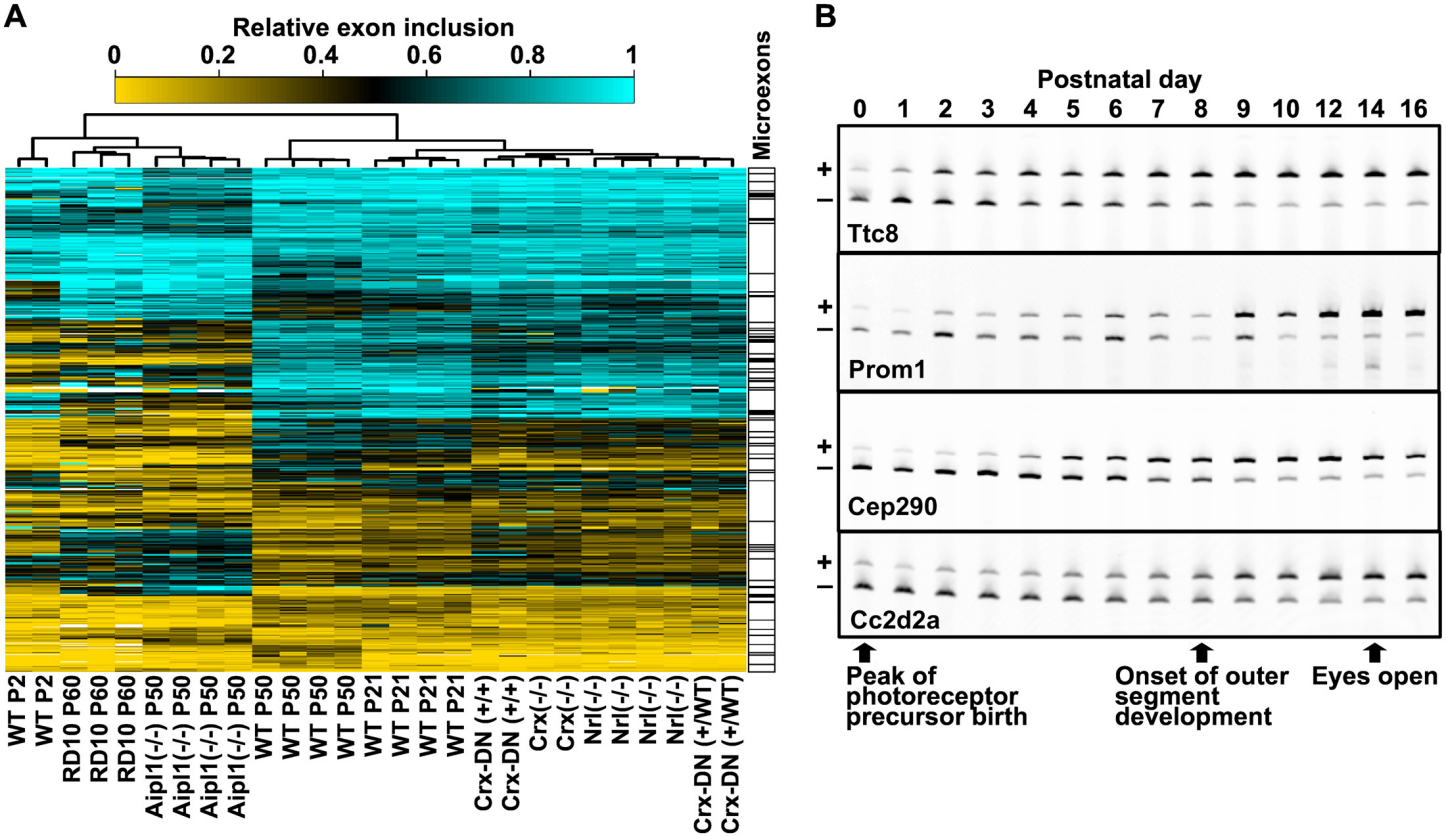
The photoreceptor splicing program is executed in the postmitotic progenitors independent of Crx. **(A)** Heatmap showing the inclusion of exons differentially spliced in photoreceptors across retinal samples from wild type mice and genetically engineered mouse models. Exon in transcripts that are not expressed in the majority of the samples are not included in the heatmap. Missing data points are in white color. Unsupervised hierarchical clustering groups the samples in two major clusters. One cluster groups samples from retinas that lack photoreceptors (RD10, Aipl1(-/-)) with wild type retina at postnatal day 2 (P2). The second cluster contains wild type retina samples from postnatal days 50 (P50) and 21 (P21) along with samples from mice carrying a dominant negative mutation in the Crx gene (Crx-DN) and knockout animals lacking the Crx or Nrl genes. Microexons with length of 30nt or less are annotated on the right. **(B)** RT-PCR analysis of the splicing of four photoreceptor specific exons in the developing retina between postnatal days 0 and 16. The bands corresponding to the exon skipped and exon included mRNA isoforms are labeled with ‘+’ and ‘-’, respectively. Key landmarks in eye development between postnatal days 0 and 16 are indicated with arrows below the gel images.

Unsupervised hierarchical clustering of the exons differentially spliced in photoreceptors revealed two major clusters (Fig 3A). One cluster is formed by samples derived from the Aipl1 knockout and the RD10 mutant retinas, both devoid of photoreceptor cells, and includes the postnatal day 2 retina samples. A second cluster is formed by the adult wild type retina, the Nrl and Crx knockouts, and the Crx-DN mutant. This clustering of the Crx knockout and Crx-DN mutant, which do not form mature photoreceptors, with the wild type retina shows that the alternative splicing in photoreceptors is controlled independently of the known transcriptional regulators of photoreceptor morphogenesis.

At postnatal day 2 the rod photoreceptors are at the stage of immature progenitors. Interestingly, the splicing profile of the postnatal day 2 retina does not cluster with the samples containing undeveloped photoreceptors that are derived from Crx knockout and Crx-DN retinas at postnatal day 21. The segregation of the postnatal day 2 retina from the juvenile Crx knockout and Crx-DN retina suggests that the photoreceptor splicing program is established in the post-mitotic photoreceptor progenitors prior to morphogenesis of the outer segment. To characterize the temporal control of alternative splicing during photoreceptor differentiation we analyzed by RT-PCR the inclusion levels of four photoreceptor specific exons in the Ttc8, Prom1, Cep290 and Cc2d2a genes between postnatal days 0 and 16 (Fig 3B). All four exons showed low levels of exon inclusion between postnatal day 0 and postnatal day 2. The inclusion levels of the four exons steadily increase thereafter, reaching half maximum at postnatal day 8, when the photoreceptor outer segments begin to develop. Thus, the shift towards photoreceptor specific isoform expression is initiated in the postmitotic photoreceptor progenitors in advance of the final stages of photoreceptor cell morphogenesis [32–34].

### Motifs for several RNA binding proteins are enriched in proximity to exons differentially spliced in photoreceptors

To determine if a specific subset of splicing regulators bind in proximity to the exons differentially spliced in photoreceptors we performed motif enrichment analysis. For this purpose we used the position weight matrices (PWM) from the Cis-BP-RNA database that describe the sites recognized by RNA binding proteins [35]. As these matrices are derived by aligning 7-mers, they fail to represent the true binding site for certain RNA binding proteins that recognize significantly shorter, 3 to 4 nucleotides long, sequences. To correct this deficiency we substituted the matrices for PTBP, NOVA, MBNL and MSI proteins with matrices corresponding to the sequences recognized by their RNA binding domains, i.e. YCU/UCY for PTBP, YCAY for NOVA, YGCY for MBNL, and UAG for MSI1.

**Fig 4:**
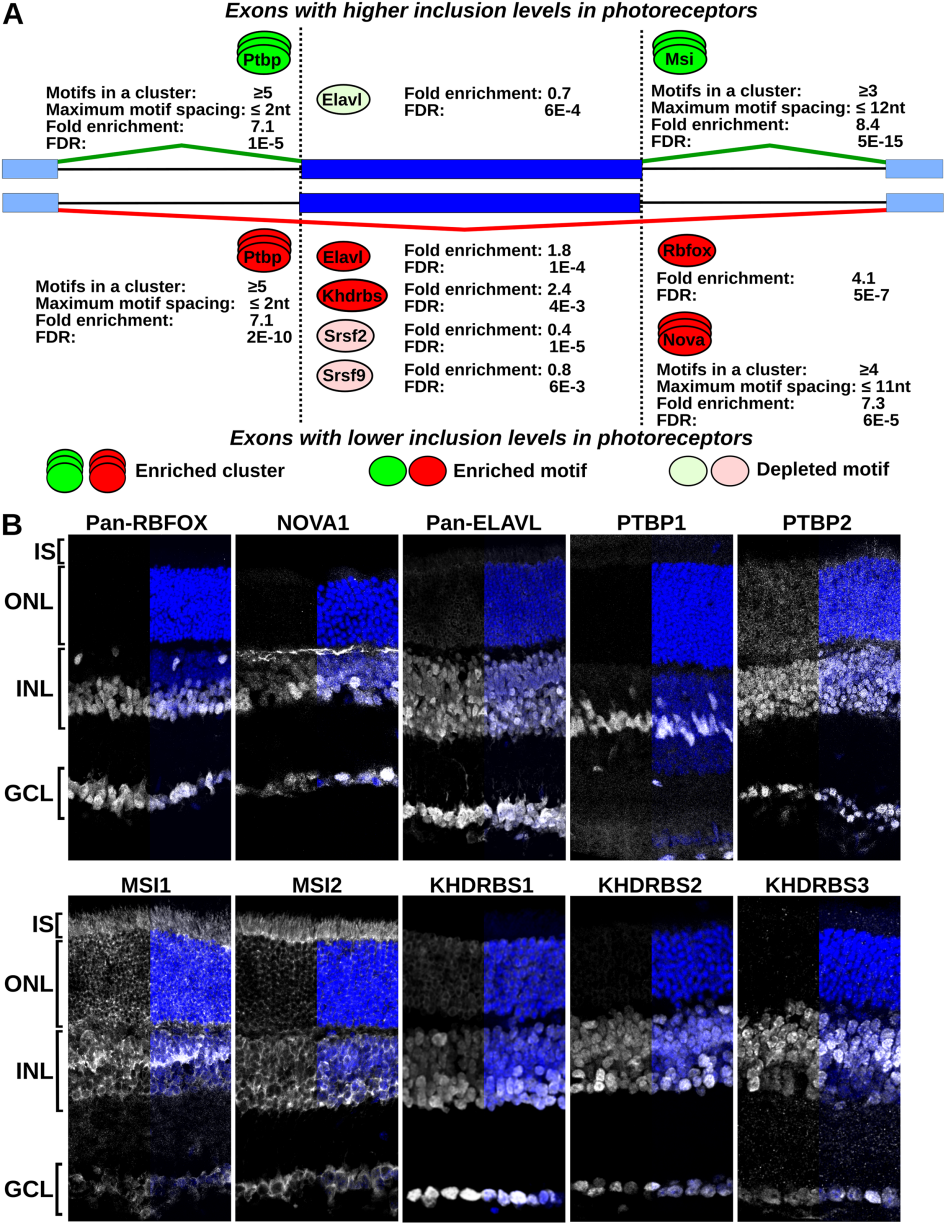
Enriched motifs for RNA binding proteins in exons differentially spliced in photoreceptors and expression of neuronal splicing regulators in the retina. **(A)** Diagram showing the position and enrichment of RNA binding protein motifs in proximity to the exons that are differentially spliced in photoreceptors. Sites enriched/depleted in the upregulated exons are shown on top in green and sites enriched/depleted in downregulated exons are shown below the exon diagram in red. Stacked ovals indicate clusters of binding sites. Pale colors indicate depletion of the motif. The fold enrichment and false discovery rate corrected p-values are shown next to each site. Clustered motifs are also labeled with the minimum number of motifs per cluster and the maximum spacing between the motifs in the cluster. **(B)** Immunofluorescence (IF) staining of retinal sections with antibodies against RBFOX, NOVA1, ELAVL, PTBP1, PTBP2, MSI1, MSI2, KHDRBS1, KHDRBS2 and KHDRBS3. IF signal is shown in grayscale and DAPI staining of the nuclear DNA is in blue. DAPI staining, shown for half of each section, indicates the position of the outer nuclear (ONL), inner nuclear (INL) and ganglion cell (GCL) layers.

Intronic sequences surrounding the differentially spliced exons showed enrichment of binding sites for several RNA binding proteins compared to the sequences surrounding exons whose inclusion levels were the same in wild type and Aipl1 knockout retina (Fig 4A and S5 Table). We observed enrichment of RBFOX and EIF2S1 binding sites, and marginal, but statistically significant enrichment of Nova binding sites downstream of exons with lower inclusion levels in photoreceptors. The enrichment of binding sites for the cytoplasmic EIF2S1 protein is likely due to similarity of the sequence it recognizes (WGCAUG) to the binding site of the RBFOX splicing factors (UGCAUG). Weak, but statistically significant enrichment of PTBP binding sites was observed upstream of all differentially spliced exons, regardless if they were included at higher rate or skipped at higher rate in photoreceptors compared to inner neurons. Musashi binding sites were enriched downstream of exons with higher inclusion levels in photoreceptors. ELAVL binding sites were partially depleted in exons with higher inclusion levels in photoreceptors and enriched in exons with lower inclusion levels in photoreceptors. Exons that had lower inclusion levels in photoreceptors showed enrichment of binding sites recognized by the KHDRBS, A1CF, LIN28, MEX3 and RBM41 proteins, all of which bind to A/U rich sequences. Binding sites for two SR proteins, SRSF2 and SRSF9, which recognize G/A rich sequences, were depleted in these exons.

Sequence elements that efficiently recruit the PTBP, NOVA, MBNL and MSI proteins typically contain clusters of the short sequences recognized by their RNA binding domains [36–40]. Thus, we tested if a higher number of PTBP, NOVA, MBNL and MSI motifs are found in clusters located in proximity to the exons that are differentially spliced in photoreceptors. PTBP binding sites in clusters containing at least 5 motifs spaced by less than 2 nucleotides were significantly enriched upstream of all differentially spliced exons (Fig 4A, S3 and S4 Figs). Such clustering of PTBP motifs is consistent with the well characterized mode of binding of the PTBP proteins to RNA. MSI binding sites in clusters containing at least three UAG motifs spaced by 10 to 15 nucleotides were enriched up to 8 fold downstream of the exons with elevated inclusion levels in photoreceptors (Fig 4A). Similar enrichment of pairs of MSI binding sites, albeit with larger spacing, was previously reported in the 3’ UTRs of transcripts whose stability and translational efficiency is controlled by MSI [38]. NOVA binding sites were also enriched in clusters downstream of exons with lower inclusion levels in photoreceptors compared to inner neurons. Overall, the motif enrichment analysis suggests a potential role in photoreceptors for several neuronal splicing regulators: RBFOX, NOVA, PTBP, KHDRBS and ELAVL.

### Neuronal splicing factors are downregulated in photoreceptors

In an attempt to identify splicing factors specific to photoreceptors we examined the expression of 1039 known and potential splicing regulators in the panel of mouse retinal samples used in our splicing analysis (S6 Table). We were unable to identify a gene that is specifically expressed in the samples with high inclusion of photoreceptor-specific exons. We observed that several key regulators of alternative splicing in neurons, Rbfox, Nova and Elavl family members, Ptbp1, Khdrbs2, and Srrm4 are downregulated in the wild type retina compared to the Aipl1 knockout (Table 1 and S1 Table).

**Table 1:** Expression level difference of neuronal splicing regulators in wild type retina compared to retina from Aipl1(-/-) mice. Significant differences in gene expression are shown in bold typeface.

*Entrez GeneID*	*Symbol*	*Log(2) Fold Change WT/AIPL1(-/-)*	*FDR*
**15568**	**Elavl1**	**-0.56**	**6.113E-3**
**15569**	**Elavl2**	**-1.21**	**1.226E-7**
**15571**	**Elavl3**	**-1.51**	**1.762E-16**
**15572**	**Elavl4**	**-1.23**	**1.796E-11**
20218	Khdrbs1	-0.16	5.493E-1
**170771**	**Khdrbs2**	**-1.33**	**5.649E-16**
13992	Khdrbs3	0.47	1.224E-2
**17690**	**Msi1**	**0.50**	**9.849E-3**
76626	Msi2	0.36	6.597E-2
**664883**	**Nova1**	**-1.69**	**3.542E-10**
**384569**	**Nova2**	**-2.02**	**1.272E-14**
**19205**	**Ptbp1**	**-0.49**	**8.269E-4**
56195	Ptbp2	-0.34	1.634E-1
230257	Ptbp3	-0.24	2.717E-1
**268859**	**Rbfox1**	**-1.32**	**1.798E-16**
**93686**	**Rbfox2**	**-1.21**	**3.027E-15**
**52897**	**Rbfox3**	**-1.20**	**1.954E-10**
**68955**	**Srrm4**	**-1.35**	**3.802E-6**

We used immunofluorescence staining to characterize the distribution of RBFOX, NOVA, PTBP, KHDRBS, and ELAVL proteins in the mouse retina (Fig 4B). We were unable to test SRRM4 due to the lack of antibodies suitable for immunofluorescence. In agreement with the RNA-Seq data, RBFOX and NOVA1 proteins were not expressed in the photoreceptor cells`. The RBFOX and NOVA proteins act as splicing activators when bound downstream of alternative exons [37,41]. Thus, the lack of RBFOX and NOVA expression in photoreceptors is consistent with the enrichment of their binding sites downstream of the exons with lower inclusion levels in photoreceptors compared to inner neurons (Fig 4A).

The PTBP proteins show the expected expression pattern with PTBP1 present in the nuclei of Mueller glia cells, while PTBP2 is expressed in the neurons and photoreceptors (Fig 4B). The absence of PTBP1 from the retinal neurons and photoreceptors releases the splicing of alternative exons carrying PTBP binding sites within the upstream intron. These exons can then be included at different level depending on the cell type, explaining the enrichment of PTBP1 binding sites upstream of exons that can be either up- or down- regulated in photoreceptors compared to inner neurons.

The ELAVL (Hu) proteins are expressed throughout the retina, with lower levels in photoreceptors, consistent with the expression differences determined by RNA-Seq (Fig 4B).

The KHDRBS family of RNA binding proteins includes the ubiquitously expressed KHDRBS1 (Sam68), and two orthologues, KHDRBS2 (Slm1) and KHDRBS3 (SLM2, T-STAR), which in the CNS are expressed in neurons [17,19,42]. Consistent with its ubiquitous expression, KHDRBS1 can be detected thought the retina. In contrast, the KHDRBS2 and KHDRBS3 proteins were expressed only in the neurons of the inner retina, but not in photoreceptors. The KHRDBS3 protein expression is most likely suppressed posttranscriptionally as the KHDRBS3 mRNA levels are uniform throughout the retina (Table 1). Accordingly, the 3'-UTR of KHDRBS3 contains conserved binding sites for microRNAs from the miR-96/miR-182/miR-183 cluster which is expressed in photoreceptors (S5 Fig) [43]. The enrichment of KHDRBS binding sites in the downregulated exons is consistent with at least one previous report showing that KHDRBS3 binds to exonic splicing enhancers to activate exon inclusion [44].

### The Musashi proteins directly regulate splicing

**Fig 5:**
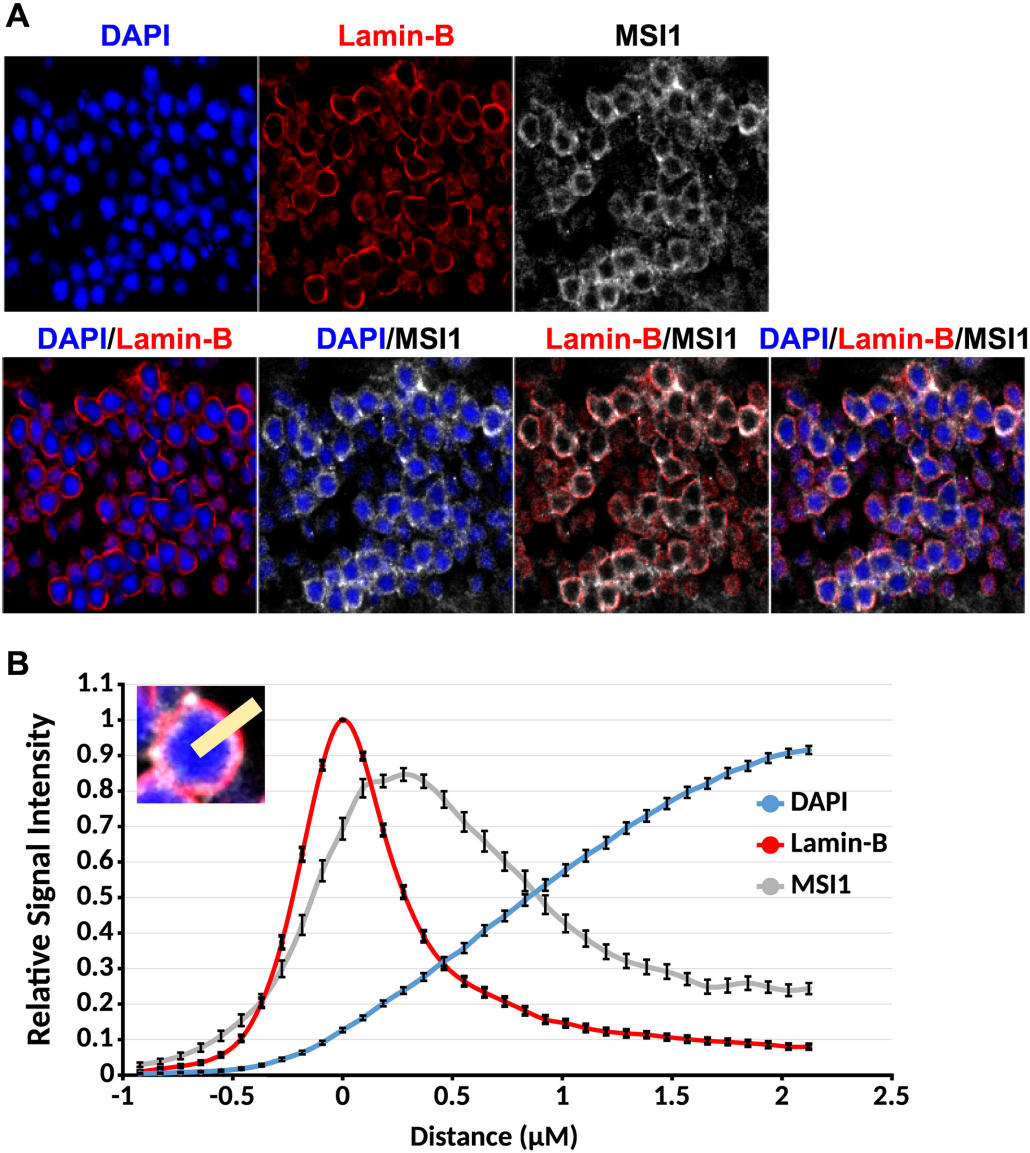
Musashi 1 is present in the nuclei of photoreceptor cells. **(A)** Immunofluorescence staining of the outer nuclear layer on 4μm retinal sections. The nuclear envelope is stained with Lamin-B antibody (red). MSI1 staining is shown in gray. The nuclear DNA is stained with DAPI (blue). **(B)** Quantification of the Lamin-B, MSI1, and DAPI signal in the nuclei of photoreceptor cells. Lamin-B, MSI1, and DAPI fluorescence intensities were measured along a line perpendicular to the nuclear envelope (inset). The intensities measured on 53 nuclei were normalized and aligned to the peak of the Lamin-B staining.

MSI binding site enrichment in the downstream intron is associated with increased inclusion levels of the alternative exons in photoreceptors. The MS1 and MSI2 proteins are expressed throughout the retina and consistent with previous reports show mostly cytoplasmic localization in the inner neuronal layers (Fig 4A) [45–47]. As an adaptation to low light environment, the heterochromatin of mouse rod photoreceptors is packed in the center of the nucleus and the nucleoplasm is pushed to the periphery [48,49]. This morphology makes DNA staining unsuitable for identifying the boundaries of the nucleus. Thus, to determine if the Musashi proteins are present in the nuclei of photoreceptors, where they can regulate splicing, we decorated the nuclear envelope with anti-Lamin antibody (Fig 5A and S6 Fig). The Lamin staining of 4μm retinal sections showed that MSI1 (Fig 5) and to lesser degree MSI2 (S6A Fig) are present in the nuclei of photoreceptor cells, where they are located in the periphery and are excluded from the heterochromatin core. We also examined the localization of the Musashi proteins in the photoreceptors of Nrl(-/-) mice. The nuclear morphology of the photoreceptors of these mice makes it easier to distinguish the nuclear and peri-nuclear compartments by fluorescent microscopy. Similar to the wild type retina, high levels of MSI1 protein were observed in the nuclei of photoreceptor cells (S6B Fig). MSI2 can also be detected in the photoreceptor nuclei, albeit its levels are higher in the cytoplasm (S6C Fig).

**Fig 6:**
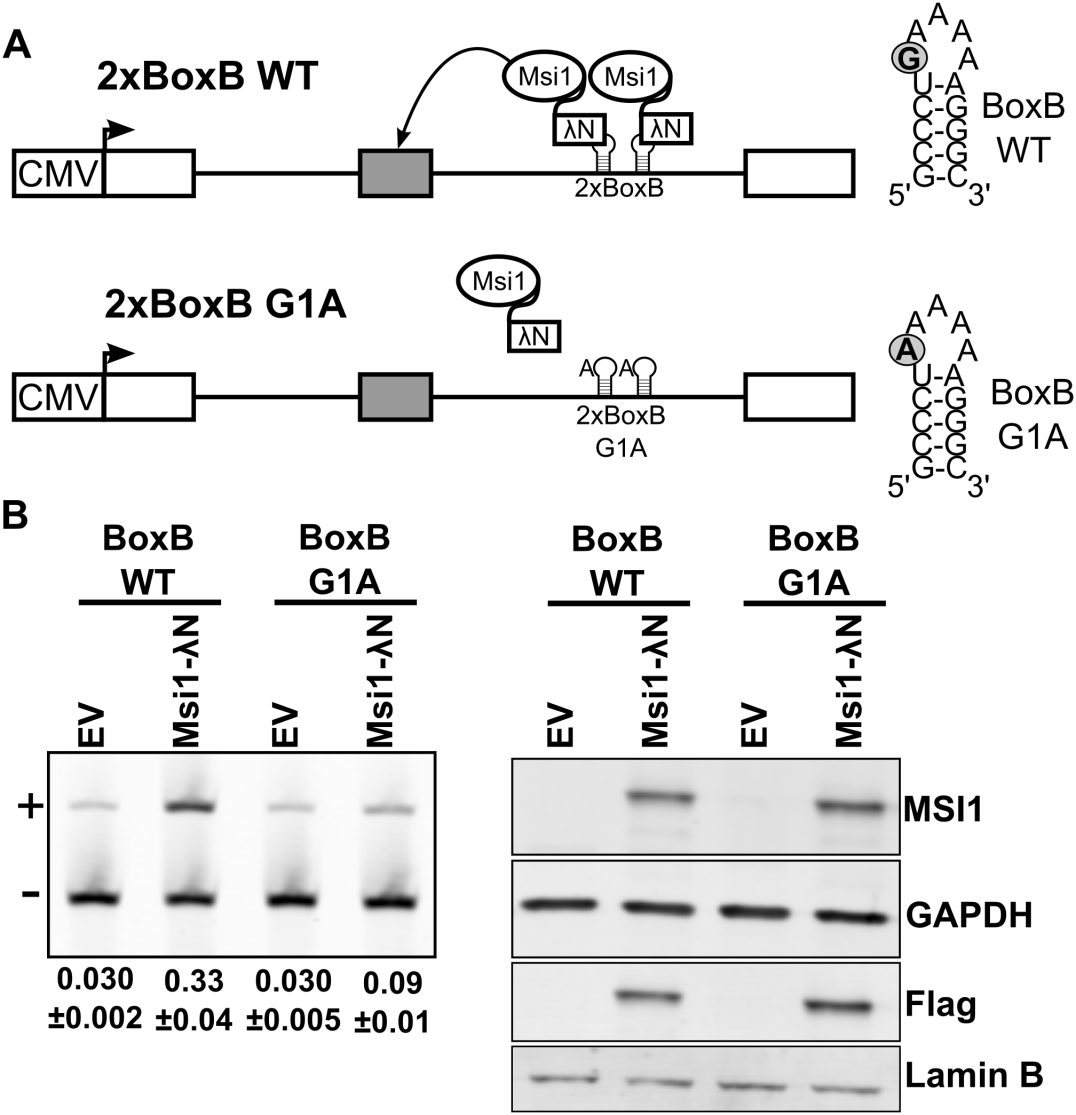
Binding of Musashi 1 downstream of an alternative exon promotes its inclusion. (A) Diagram of the BoxB minigenes. In the wild type minigene two BoxB hairpins are positioned in the intron downstream of an alternative exon. In the G1A minigene a G to A mutation in the BoxB hairpins prevents binding of the λN peptide to the RNA. (B) RT-PCR analysis of the splicing of the wild type and G1A minigenes after co-transfection with empty vector (EV) or vector expressing MSI1-λN fusion (left). The exon included and exon skipped isoforms are indicated with ‘+’ and ‘-’, respectively. Relative exon inclusion levels with standard error are shown below each lane. On the right, western blot shows the expression levels of the MSI1-λN protein as detected by the anti-Msi1 and anti-Flag antibodies. GAPDH and Lamin-B are used as loading controls.

To test if the Musashi proteins can promote inclusion of an alternative exon when bound downstream of it we used a splicing reporter that has two lambda phage BoxB RNA hairpins engineered downstream of an artificial alternative exon [50]. The loop of the BoxB hairpin is specifically bound by the lambda N-peptide. Consequently, proteins tagged with the lambda N-peptide are tethered to the BoxB elements on the reporter pre-mRNA. Cotransfection of the reporter with Musashi lambda-N fusions increased inclusion of the reporter exon (Fig 6 and S7A Fig). The effect of Musashi on splicing is completely abolished in a reporter containing G to A point mutations in the two BoxB elements that disrupt binding of the lambda-N peptide. To test if the effect of MSI1-lamda-N fusion is specific we cotransfected the BoxB reporter with lambda-N fusions for three well characterized splicing factors, PTBP1, SRSF4 and TRA2B. None of these splicing factors increased the inclusion level of the test exon when tethered downstream of it, demonstrating that this is an effect specific to the MSI1 protein (S7C Fig).

### The Musashi proteins promote Ttc8 exon 2A inclusion in photoreceptors

**Fig 7:**
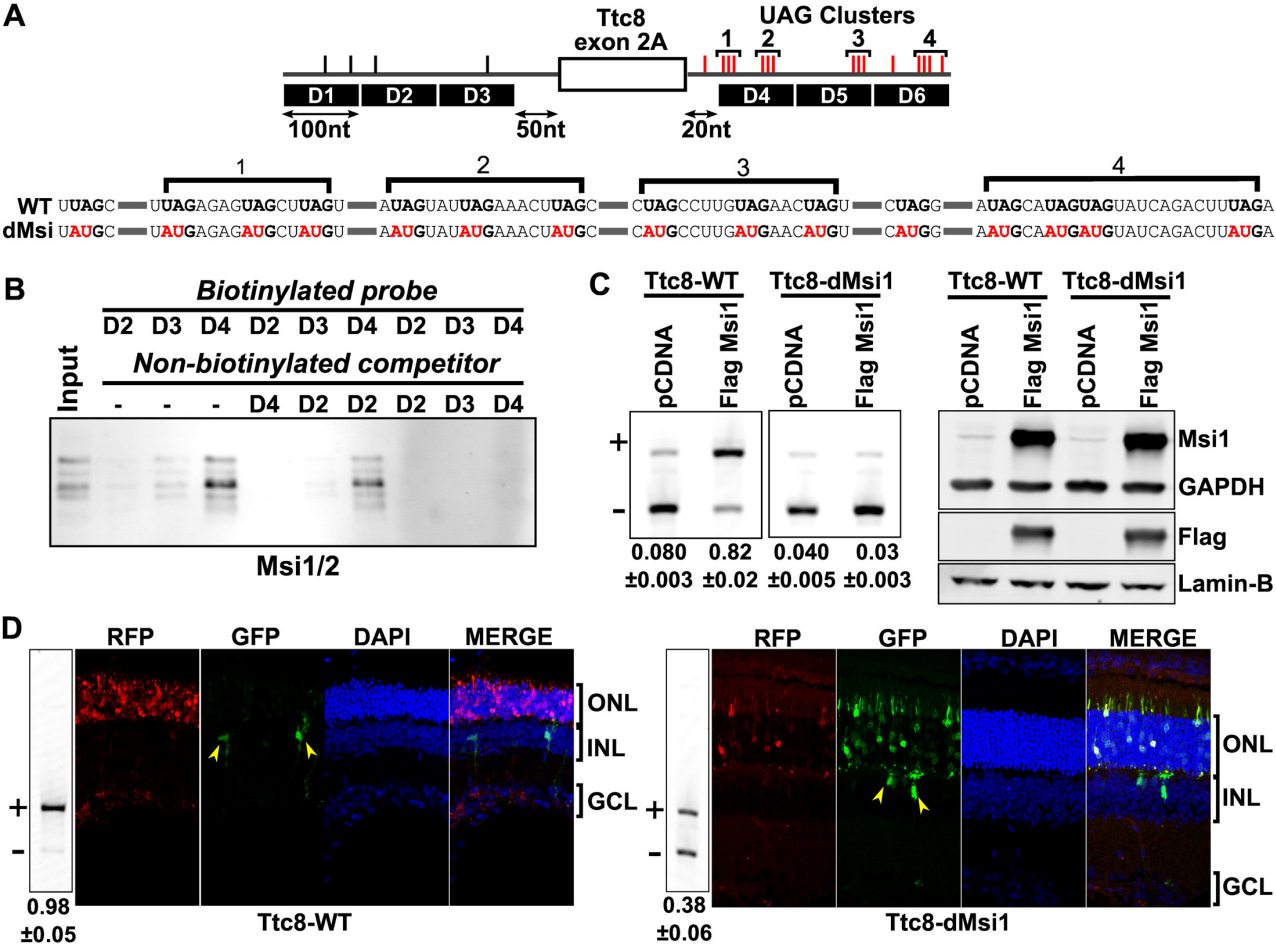
Musashi proteins bind downstream of Ttc8 exon 2A and promote its inclusion in photoreceptor cells. **(A)** Diagram of Ttc8 exon 2A and the adjacent introns. Musashi binding sites in the introns are shown with tick marks above the intron. Clusters of Musashi binding sites in the downstream intron are labeled with numbers. Binding sites in the downstream intron that were mutated to create the Ttc8-dMsi minigene are shown in red. The sequence mutated in the Ttc8-dMsi minigene is shown below the diagram with the mutated bases in red. **(B)** Pull-down of Musashi proteins from retinal extracts. Three biotinylated probes corresponding to segments D2, D3, and D4 were used to pull down RNA binding proteins from retinal extracts. The binding was competed with the competitor RNA as indicated above. The Musashi proteins were then detected by western blot using an antibody that recognizes both MSI1 and MSI2. **(C)** RT-PCR analysis of the wild type and mutant Ttc8 exon 2A minigene transcripts after co-transfection with construct expressing Flag-tagged MSI1 protein. The exon included and exon skipped isoforms are indicated with ‘+’ and ‘-’, respectively. Relative exon inclusion levels with standard error are shown below each lane. On the right, western blot shows the expression levels of the MSI1-Flag protein as detected by the anti-MSI1 and anti-Flag antibodies. GAPDH and Lamin-B are used as loading controls. **(D)** RT-PCR analysis and fluorescence imaging of the splicing of the Ttc8 minigene transcripts in the retina. Mouse retinas were electroporated with each minigene at postnatal day 0 and the splicing was analyzed by RT-PCR at postnatal day 16 and by fluorescence imaging at postnatal day 20. Relative exon inclusion levels with standard error are shown below each lane. The minigene is designed to produce GFP when the alternative exon is skipped or RFP when the exon is included. The wild type minigene is shown on the right. High RFP and low GFP expression in the photoreceptors indicates that the exon is included in the mature transcripts from the minigene. The inner neurons, marked with yellow arrows, express almost exclusively GFP, an indication that the exon is skipped. Reduced RFP expression and increased GFP levels in the mutant minigene indicate that the exon is mostly skipped in photoreceptors, in agreement with the RT-PCR analysis.

To determine if the Musashi proteins regulate splicing in photoreceptors we turned to the photoreceptor specific exon 2A in the Ttc8 (Bbs8) gene. We previously mapped two 100nt sequence segments in the introns immediately adjacent to exon 2A that act in concert to promote the splicing of this exon in photoreceptors [23]. Deletion mutagenesis showed that these segments contain multiple redundant cis-acting sequences [23]. The D4 segment located immediately downstream of exon 2A carries two clusters of Musashi binding sites, each containing three UAG motifs (Fig 7A). Within 320nt of the downstream intron immediately adjacent to exon 2A we find two more clusters containing three and four UAG motifs, respectively. In contrast, the 350nt section of the intron immediately upstream of exon 2A contains four Musashi binding sites, approximately the number of UAG triples that would be expected in random sequence of this size. To determine if the Musashi proteins can bind specifically to the D4 segment we used biotinylated RNA corresponding to this element to pull-down RNA binding proteins from retinal extracts. We also performed the pull-down with the other regulatory element, D3, and with segment D2, which is not required for splicing of exon 2A in photoreceptors. The binding was competed with non-biotinylated RNA, either of the same sequence or different sequence of the same length. In this pulldown experiment the Musashi proteins bind specifically to segment D4 (Fig 7B). In contrast, segments D2 and D3, each of which contains a single UAG motif, had low affinity for the Musashi proteins and the binding was completely blocked by competitor RNA.

To determine how MSI1 binding downstream of exon 2A affects its inclusion levels in the retina we used a reporter minigene designed to produce GFP when the exon is skipped and RFP when the exon is included [23,51]. We mutated all 15 Musashi binding sites in the downstream intron of the minigene (Fig 7A). Both the wild type and mutant minigenes were co-transfected with MSI1 expression construct in N2A cells. MSI1 promoted the inclusion of the wild type exon 2A but had no effect on the mutant minigene (Fig 7C and S7A Fig). To determine if the MSI binding sites are required for splicing of Ttc8 exon 2A in photoreceptor cells, we electroporated the wild type and mutant minigenes in the retina of neonate mice. We allowed the photoreceptors to develop and analyzed the splicing of the minigene transcripts in the retina by RT-PCR and immunofluorescence at postnatal days 16 and 20, respectively. As we have shown previously, the wild type Ttc8 exon 2A is included at high levels (98%) in the photoreceptors and is excluded from the mature transcripts in the inner neurons (Fig 7D and S7B Fig) [23]. Consistent with a role for MSI1 in directing splicing in photoreceptors, the inclusion level of exon 2A in the transcripts of the mutant minigene was reduced to approximately 38%.

### MSI1 promotes the inclusion of photoreceptor specific exons

**Fig 8:**
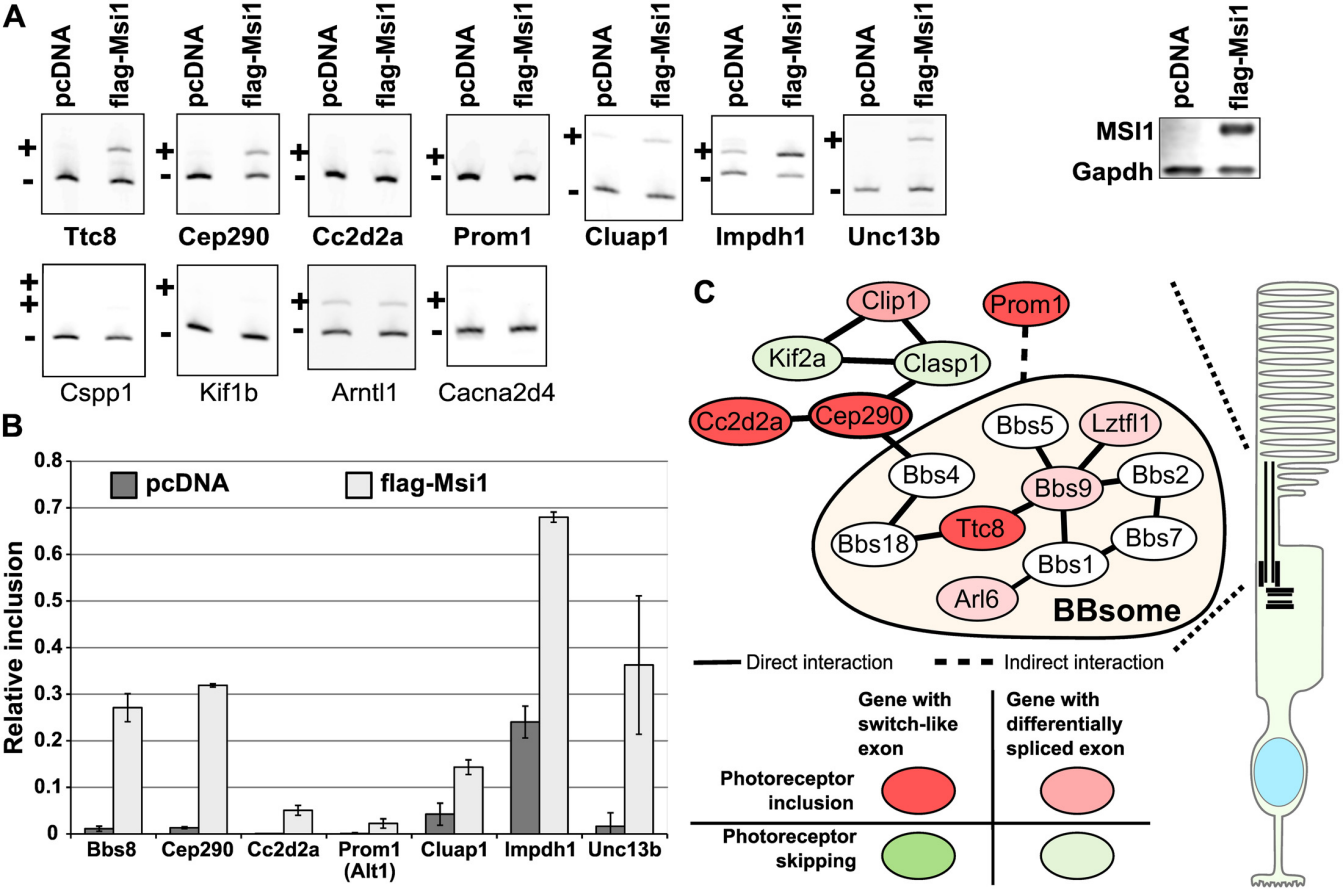
Musashi 1 promotes the inclusion of photoreceptor specific exons. **(A)** RT-PCR analysis of the inclusion levels of 11 exons with elevated inclusion levels in photoreceptors in N2A cell transfected with Flag-tagged MSI1 expression construct. The exon included and exon skipped isoforms are indicated with ‘+’ and ‘-’ respectively. On the right, western blot shows the expression of the flag-tagged Msi1 protein. **(B)** Chart showing the quantification of the inclusion levels of seven exons affected by the Msi1 protein. **(C)** Diagram showing a protein interaction network enriched in photoreceptor specific isoforms. The network is centered on Cep290 and the BBsome. Genes containing “switch-like” exons are shown in dark red or green. Red and green colors indicate inclusion and skipping in photoreceptors, respectively. Genes containing exons differentially spliced in photoreceptors are shown in pale red or green.

The enrichment of Musashi binding sites downstream of exons with elevated inclusion levels in photoreceptors suggests that multiple alternative exons should be regulated by the Musashi proteins in addition to Ttc8 exon 2A. To test this prediction, we expressed flag-tagged MSI1 in N2A cells and analyzed by RT-PCR the splicing of eleven endogenous transcripts containing exons with elevated inclusion levels in photoreceptors. MSI1 caused statistically significant increase in the inclusion levels of seven of the eleven exons (Fig 8). Inclusion levels of five of these exons, including Ttc8 exon 2A of the endogenous Ttc8 gene, increased by at least 10% in response to MSI1 expression (Fig 8). The smaller amplitude of the effect of Msi1 transfection on the inclusion levels of the endogenous Ttc8 exon 2A compared to the minigene transcripts is likely due to the transfection efficiency, which was approximately 40% in these experiments. Among the exons coordinately regulated by the MSI1 are four “switch-like” exons in Ttc8, Cep290, Cc2d2a and Prom1. All four genes encode ubiquitously expressed proteins that are involved in ciliary biogenesis and function. Ttc8, Cep290, Cc2d2a and Prom1 are also required for the development and maintenance of the photoreceptor outer segments [52–54].

## Discussion

The lack of comprehensive gene expression profiles of defined neuronal subtypes is a major obstacle to understanding how the neuronal diversity of the vertebrate CNS is established. To delineate the gene expression and alternative splicing programs of a single neuronal subtype we turned to the vertebrate retina. The high abundance of rod photoreceptors in the mouse retina allowed us to isolate the characteristic features of their transcriptome by comparing the Aipl1 knockout model of retinal degeneration to wild type mice.

The deduced splicing profile of photoreceptor cells is related to the spicing profiles of retinal and CNS neurons (Fig 2 and S1 Fig). Alternative splicing in neurons is known to be regulated by SRRM4 and members of the PTBP, RBFOX, KHDRBS, NOVA and ELAVL families of RNA binding proteins [14,16–19]. Strikingly, photoreceptors do not express RBFOX, NOVA, KHDRBS2 and KHDRBS3 proteins, express lower amounts of ELAVL proteins and have markedly lower Srrm4 transcript levels. In light of these data, the switch from PTBP1 to PTBP2 expression during development emerges as a major determinant of the alternative splicing program that is shared between neurons and photoreceptor cells [55,56].

In the absence of a “master” regulator of splicing specific to photoreceptors, the characteristic splicing program of these cells is likely determined by a unique combination of splicing factors with broader expression.

We show that Musashi 1 (MSI1) promotes the splicing of exons with elevated inclusion levels in photoreceptors. The Musashi proteins are notable for their expression in stem cells, where they are involved in stem cell maintenance and cell fate determination [57–59]. The best characterized function of Musashi is the regulation of mRNA stability and translation through binding to the 3’-UTR of the target transcripts [58]. In the retina, the subcellular localization of the Musashi proteins varies during development and in mature neurons the two proteins are confined to the cytoplasm (Fig 4) [45]. Here we show that MSI1 is present not only in the cytoplasm but is also abundant in the nuclei of photoreceptor cells, where it controls alternative pre-mRNA splicing. Our findings contrast recent studies of the Musashi activity in the central nervous system and glioblastoma, where the cytoplasmic localization of the Musashi proteins confines their function to control of mRNA stability and translation and limits their impact on splicing [60,61]. Control of the subcellular localization of MSI1 provides a mechanism that can produce the characteristic splicing program of photoreceptors in the absence of a photoreceptor-specific RNA binding protein. Similar mechanisms that involve redistribution of RNA binding proteins between the nucleus and the cytoplasm are known to control alternative splicing in response to external stimuli [62–64]. While playing a significant role in promoting inclusion of photoreceptor specific exons, MSI1 is clearly not the sole determinant of the photoreceptor specific splicing program. The absence from photoreceptors of the RBFOX, NOVA and KHDRBS2/3 proteins also contributes to the differences in alternative splicing between photoreceptors and neurons as indicated by our motif enrichment analysis. Our results suggest that the characteristic splicing program of the photoreceptor cells may be determined by a unique suite of splicing factors with broader expression that can combinatorically form different exon recognition complexes depending on the sequence of the underlying RNA substrate. Such combinatorial control of splicing is a well-established paradigm and was recently demonstrated to control "switch-like" splicing events during reprograming of primary fibroblasts into pluripotent stem cells [65,66]. Further research will be needed to directly demonstrate the combinatorial control of the inclusion of photoreceptor specific exons and identify the factors beyond MSI1 that are involved in this process.

At present it is unclear how most of the alternative exons we identified affect protein function or to what degree alternative splicing shapes the properties of the photoreceptor cells. One exception is the 14nt exon 8 in the Arl6 (BBS3) gene, which has high inclusion levels in photoreceptors (Fig 1). ARL6, a Ras family GTP-binding protein, is part of a network of proteins involved in the development and maintenance of primary cilia (Fig 8C). The exon 8 containing isoform of ARL6 is required for normal vision in zebra fish [20,21]. The vision phenotype of mice lacking Arl6 exon 8 has not been reported in detail, however gross histological examination and immunofluorescent staining of the retina show that the inner segments of photoreceptors are disorganized [21]. Several other components of the protein network that ARL6 is part of are also differentially spliced in photoreceptors. Four of these genes, Cep290, Cc2d2a, Ttc8 and Prom1 contain “switch-like” exons that produce isoforms highly specific to photoreceptors (Figs 8C and 1B) [22,23]. Strikingly, the splicing of these “switch-like” exons is coordinately regulated in development (Fig 3B) and their inclusion is promoted by MSI1.

The photoreceptor splicing program is activated in the postmitotic progenitors, prior to the onset of outer segment development. A transcription factor cascade starting from Crx homeobox protein that is critical for photoreceptor morphogenesis is also activated during the same developmental time frame [31,67,68]. Interestingly, alternative splicing in photoreceptors is not affected in the Crx knockout animals and in the Crx dominant negative mutant [29]. Thus, the developmental switch to photoreceptor specific splicing is independent of the established transcriptional mechanism that activates the expression of photoreceptor specific genes.

In summary, we demonstrate that photoreceptors express a characteristic splicing program that encompasses hundreds of alternative exons and affects the transcripts of multiple genes that are critical for vision.

## Materials and Methods

### Ethics statement

All procedures carried out on laboratory mice are in full compliance with all federal regulations and were approved by Institutional Animal Care and Use Committee at West Virginia University.

### Clones and antibodies

Musashi 1 cDNA was amplified from mouse retinal cDNA using primers that introduced the flag-tag and cloned into pCDNA3.1 (See S10 Table for primer sequences). The PKC-neg-40B-2xBoxB-EGFP splicing reporter and pIBX-C-FF-(B)-NLS-λN expression vector were described previously [50,69]. The Ttc8 exon 2A minigene containing mutant Musashi 1 consensus binding motifs was created using Gibson Assembly (See S10 Table for the oligonucleotide sequences). Antibodies used in this work are listed in S8 Table.

### Mice

All procedures carried out on laboratory mice were approved by Institutional Animal Care and Use Committee at West Virginia University (WVU). Subretinal injection, time course analyses, and immunofluorescence of sections were performed on CD-1 mice (Charles River). Subretinal injection and electroporation of DNA was carried out on newborn CD-1 pups as described previously [70]. Toluidine blue staining was performed by Excalibur Pathology Inc. on retina sections from p65 C57bl/6j and p60 C57bl/6j:Aipl1(-/-) mice.

### RNA-Seq library preparation and sequencing

Total RNA was isolated from wild type C57bl/6j and C57bl/6j:Aipl1 (-/-) retinas at postnatal day 50 using Tri-reagent (Sigma). rRNA subtracted RNA-Seq libraries were generated using 1μg of total RNA per replicate using RiboZero and TruSeq kits (Illumina). Four replicates, each derived from different animal, were generated for each wild type and Aipl1(-/-) sample. The libraries were sequenced to a depth of 43 million reads (range 39 to 47 million reads) on Illumina Hi-Seq 15000. The reads produced by the RNA-Seq experiments are deposited at the NCBI SRA repository under accession number SRP068974.

### Bioinformatics analysis

Reads from the retinal samples were mapped to the current mouse genome (GRCm38) using TopHat. Following the mapping, Cufflinks was used to carry out guided transcriptome assembly based on the ENSEMBL GRCm38 annotation (S1 Data file contains the updated annotation in GTF format). Additional RNA-Seq data sets for mouse tissues and retinal samples from genetically engineered mouse models (Nrl knockout, Crx knockout, Crx dominant negative and RD10 mutant) were downloaded from the NCBI sequence read archive and aligned using the updated annotation. The accession numbers of the data sets not generated by us are listed in S8 Table.

Exon inclusion levels across all samples were calculated using rMATS version 3.08 [71]. We added to rMATS a basic capability to discover novel exons within annotated transcripts based on splice junction reads that are anchored on one end to a known exon (S8 Fig). rMATS was used to carry out differential splicing analysis of the wild type and Aipl1(-/-) retina samples. Differences in gene expression between the wild type and Aipl1(-/-) samples were identified using featureCounts and edgeR [72,73]. Gene Ontology analysis was carried out using WebGestalt [74].

Motif enrichment analysis was carried out in R/Bioconductor using the PWMEnriched package [75]. Position weight matrices for RNA binding proteins were described previously [35]. The matrices for RBFOX, PTBP, MBNL and MSI proteins were replaced with the matrices listed in S9 Table. Binding sites carrying at least 90% match to the scoring matrices were counted in the exons and in 200nt segments of the introns immediately adjacent to the exon. Binding sites for orthologues recognizing highly similar sequences, e.g. RBFOX1, 2 and 3 proteins, KHDRBS1, 2 and 3 proteins, etc. were pooled together. Binding that overlap by more than 50% were counted as a single site. Two single tailed hypergeometric tests were used to determine the significance of the binding site enrichment/depletion in each segment. The hypergeometric test p-values were corrected for multiple testing using Benjamini-Hohberg's procedure. To assess if there is an enrichment of clustered binding sites, the analysis for MSI, PTBP, ELAVL and NOVA was repeated after excluding the binding sites not located within a cluster. Clusters were defined by two parameters: minimum number of binding sites necessary to form a cluster, ranging from 2 to 5; and the maximum spacing between them, ranging from 0nt to 30nt. The enrichment analysis was carried out for each pair of minimum binding site count and maximum spacing parameters.

The micro-RNAs targeting Khdrbs3 were identified by microRNA.org based on the miRanda and mirSVR predictions algorithms [76–78].

### RNA isolation and RT-PCR from retina

RNA from post-natal day 16 retinas was isolated with TRI reagent (Sigma) according to manufacturer’s guidelines and reverse transcribed using mixture of random hexamers and oligo-dT to prime the cDNA synthesis. Alternatively spliced regions were amplified using fluorescently labeled primers positioned in the flanking exons (See S10 Table for primer sequences). The amplified products were separated by gel electrophoresis under denaturing conditions and imaged on a Typhoon 9410 imager (GE).

### Retinal tissue sections and fluorescence imaging

Retinal sections were prepared, stained and imaged as described previously [23]. Musashi, Lamin-B, and DAPI co-localization analysis was performed on 4μm sections using ImageJ software to plot signal intensities spanning a 10μm line perpendicular to the border of nuclei in the ONL, n = 53. Signals from individual nuclei were normalized to the maximal signal for each channel and each set of measurements were centered relative to the maximal Lamin-B signal before averaging and plotting data.

### Cell line transfections and RNA isolation

Transient transfection of N2A cells were carried out using polyethyleneimine. After 48 hours total RNA was isolated with TRI reagent (Sigma) according to manufacturer’s guidelines. To isolate total protein for western blot the cells were lysed in SDS sample buffer.

293T cells were transfected using Mirus Transit 293 reagent with 150ng PKC-neg-40B-2xBoxB-EGFP or PKC-neg-40B-2xBoxB(G1A)-EGFP and 50ng pIBX-C-FF-(B)-NLS-λN (empty vector, Msi1 or Msi2) per well of a 24 well plate in triplicate. RNA and protein were isolated 48 hours later using Trizol reagent and RIPA buffer, respectively. RT-PCR of the mini-gene was carried out using primers in the flanking exons of the 40nt test exon with the reverse primer being FAM labeled.

### Western blotting

The protein samples were resolved in 10% SDS-PAGE gel electrophoresis before being transferred to an Immobilon FL membrane (Millipore.) Membranes were blocked in Tris buffered saline solution containing 1% Tween-20 and 0.25% bovine skin gelatin. The membranes were incubated with primary antibodies overnight at in the blocking solution. After removing the primary antibody and washing the membrane in the blocking solution, the secondary antibodies were applied in the blocking solution for 1 hour. Membranes were washed again in the blocking solution and imaged on a Typhoon 9410 imager (GE) after washing with PBS.

### RNA pull-down

RNA probes were synthesized with the Hi-Scribe T7 RNA Synthesis kit (NEB) using 0.5μg of PCR amplified template DNA (See S10 Table for primer sequences). 100pmol of RNA probes were then biotinylated using the Pierce RNA 3’ end biotinylation kit (Thermo Fisher) and purified according to manufacturer’s instructions. Biotinylated RNA probes were re-suspended in 100μl of high salt buffer (0.5M NaCl, 10mM Hepes pH7.9). Approximately 0.4mg of streptavidin magnetic beads (NEB) were washed in high salt buffer and incubated with biotinylated probes on ice for 1–2 hours with occasional mixing. The beads were then washed three times with wash buffer (0.1M KCl, 10mMHepes pH 7.9, 0.1% Triton-X100). Washed beads were incubated on ice with 100μg retinal extract and 6μg competitor RNA in binding buffer (0.1M KCl, 10mMHepes pH 7.9, 5μg/μl heparin, 0.1% Triton-X100 and 20U RNAse Inhibitor) for four hours with occasional mixing. Beads were then washed three times with wash buffer and the bound proteins were eluted in wash buffer containing 20ng RNAse A. The Musashi proteins in the eluates were detected by western blotting using an antibody that reacts with both MSI1 and MSI2 (S8 Table).

### Flow sorting of rod photoreceptor cells

Label-free isolation of rod photoreceptor cells by flow cytometry was carried out as described before (S2A Fig) [79]. The identity of the sorted cells was confirmed by quantitative RT-PCR using markers for rod photoreceptors, different inner neuron types and glial cells (S2B Fig) [80]. The expression levels were normalized to the geometric average of four reference genes: Gapdh, Hprt, Sdha and Pgk1. The primers used in the qPCR assays are listed in S10 Table.

## Supporting Information

S1 Fig Retinal neurons express a characteristic splicing program that is related to the splicing program of CNS neurons. **(A)** Heat map showing unsupervised hierarchical clustering of a panel of mouse tissues based on the inclusion levels of 8539 alternative exons. Microexons of 30nt or less in length are annotated on the right. Retinal samples form an independent cluster which is related to the cluster formed by the samples from the central nervous system and show frequent use of microexons. Exon in transcripts that are not expressed in the majority of the samples are not included in the heatmap. Missing data points are in white color. **(B)** Unsupervised hierarchical clustering of tissue samples based on the inclusion levels of 483 microexons shows elevated microexon use in neuronal tissues. A subset of the microexons marked with a red box on the left of the heat map are specifically included in retinal transcripts.
(TIF)

S2 Fig RT-PCR analysis of the inclusion levels of exons differentially spliced in wild type retina, Aipl1(-/-) retina, Nrl(-/-) retina and flow sorted rod photoreceptors. **(A)** Forward (FSC) vs Side scatter (SSC) scatter plots of dissociated retina from wild type and Aipl1(-/-) mice. The rod photoreceptor population is circled in red on the plot of the wild type retina cells. The rod population is absent from the Aipl1 knockout retina. **(B)** Quantitative RT-PCR analysis of the expression of photoreceptor, inner neuron and glial markers in the flow sorted rod photoreceptor cells, wild type retina and retina from Aipl1(-/-) mice. The expression levels of all marker genes are normalized to the levels in wild type retina. High levels of the rod transducin (Gnat1) are readily detectable in the flow sorted rod cell population, while the levels of inner neuron and glial cell markers were at or below the assay detection limits. **(C)** RT-PCR analysis of alternative splicing in wild type retina, Aipl1(-/-) retina, Nrl(-/-) retina and flow sorted rod photoreceptors. The bands corresponding to the exon skipped and exon included mRNA isoforms are labeled with ‘+’ and ‘-’, respectively. The relative exon inclusion and standard error of three independent replicates are shown below each lane. **(D)** The difference in exon inclusion between rod photoreceptor cells and whole retina inversely correlates with the expression level of the gene in photoreceptors, approximated by the fold change in the expression between wild type and Aipl1 retina. The exon inclusion and gene expression levels were determined by RT-PCR and RNA-Seq, respectively. The inverse correlation is due to the mixed cell type composition of the whole retina and illustrates a limitation in our approach that may prevent the reliable discovery of photoreceptor specific splicing variants of genes with relatively low expression levels in photoreceptors compared to inner neurons.
(TIF)

S3 Fig Enrichment of MSI, PTBP and NOVA binding site motifs in clusters adjacent to exons upregulated in photoreceptors. Clusters with minimum size of 2, 3, 4 or 5 motifs were tested for each protein. The spacing between the motifs in a cluster was varied from 0 to 30nt (X—axis). Enrichment upstream or downstream of the exons is plotted with circles and triangles, respectively. Statistically enriched clusters are represented by filled markers using red or blue colors for positions upstream or downstream of the exon, respectively.
(TIF)

S4 Fig Enrichment of MSI, PTBP and NOVA binding site motifs in clusters adjacent to exons downregulated in photoreceptors. Clusters with minimum size of 2, 3, 4 or 5 motifs were tested for each protein. The spacing between the motifs in a cluster was varied from 0 to 30nt (X—axis). Enrichment upstream or downstream of the exons is plotted with circles and triangles, respectively. Statistically enriched clusters are represented by filled markers using red or blue colors for positions upstream or downstream of the exon, respectively.
(TIF)

S5 Fig Khdrbs3 is targeted by micro-RNAs from the mir-96/182/183 cluster. **(A)** Predicted binding sites for retinal micro-RNAs in the 3'-UTR of Khdrb3. Binding sites conserved between mouse and human are shown in bold typeface. **(B)** Alignment of the retina specific micro-RNAs to the predicted binding sites. Seed sequences conserved between mouse and human are underlined. Each alignment is accompanied with mirSVR score representing the predicted efficiency of the target site (lower score means higher efficiency) and PhastCons sequence conservation score [76].
(TIF)

S6 Fig Subcellular localization of the Musashi protein in the photoreceptors of wild type and Nrl(-/-) mice. **(A)** Immunofluorescence detection of MSI2 in the outer nuclear layer of wild type mouse retina (6μm sections). The nuclear envelope is stained with Lamin-B antibody (red). MSI2 staining is shown in gray. The nuclear DNA is stained with DAPI (blue). **(B and C)** Immunofluorescence detection of MSI1 and MSI2, respectively, in the outer nuclear layer of NRL(-/-) mouse retina (6μm sections). The nuclear envelope is stained with Lamin-B antibody (red). Musashi protein staining is shown in gray. The nuclear DNA is stained with DAPI (blue). The Musashi proteins were visualized on the same section using rat -anti-MSI1 and rabbit anti-MSI2 antibodies in combination with anti-rat AF647 and anti-rabbit AF655 secondary antibodies.
(TIF)

S7 Fig Binding of the Musashi proteins downstream of an alternative exon promotes its inclusion. **(A)** RT-PCR analysis of the splicing of the wild type and G1A minigenes after co-transfection with empty vector or vectors expressing MSI1- λN and MSI2- λN fusions (top). The exon included and exon skipped isoforms are indicated with ‘+’ and ‘-’, respectively. Relative exon inclusion levels with standard error are shown below each lane. Below, western blot shows the expression levels of the MSI1- λN and MSI2- λN proteins. GAPDH and Lamin-B are used as loading controls. **(B)** RT-PCR analysis of the wild type and mutant Ttc8 exon 2A minigene transcripts after co-transfection with construct expressing flag-tagged MSI1 and MSI2 proteins. The exon included and exon skipped isoforms are indicated with ‘+’ and ‘-’, respectively. Relative exon inclusion levels with standard error are shown below each lane. Below, western blot shows the expression levels of the MSI1 and MSI2 proteins. Lamin-B is used as loading control. **(C)** RT-PCR (agarose gel on the left) shows that the lambda-N fusion of MSI1 but not PTBP1, SRSF4 and TRA2B, is capable of increasing the inclusion of the test exon when tethered downstream of it. Western blot (right), shows abundant expression of all four fusions. The expected band for each fusion is indicated by an asterisk.
(TIF)

S8 Fig New exon discovery within MATS. Junction reads that map on one end of an exon in annotated transcripts are used to identify novel exons. A novel exon is defined by two sets of junction reads of at least 10 reads per set, one anchored on the left and a second one anchored on the right to a known exon, that map within a predefined distance (300nt) from each other.
(TIF)

S1 Table Differential gene expression in WT vs AIPL1 knockout retina. (XLSX)

S2 Table GO categories enriched in genes differentially expressed in wild type compared to Aipl1(-/-) retina. (XLSX)

S3 Table Alternative splicing analysis. Exons with significant difference in the inclusion levels in wild type retina compared to the Aipl1 knockout are highlighted.
(XLSX)

S4 Table Summary of GO terms enriched in genes with exons differentially spliced in wild type retina compared to retina from Aipl1(-/-) animals. (XLSX)

S5 Table Summary of binding sites with significant enrichment in the regulated exons and/or 200nt of the adjacent introns. (XLSX)

S6 Table List of known and potential splicing regulators. (XLSX)

S7 Table Antibodies used in this work. (XLSX)

S8 Table RNA-Seq datasets not generated by this study. (XLSX)

S9 Table Position weight matrices for Ptbp, Mbnl, Msi and Rbfox. (XLSX)

S10 Table Primers used in this work. (XLSX)

S1 Data Updated transcriptome annotation in GTF format. (GZ)
