# Correction: The Musashi 1 Controls the Splicing of Photoreceptor-Specific Exons in the Vertebrate Retina

**DOI:** 10.1371/journal.pgen.1006432

**Published:** 2016-11-03

**Authors:** PLOS Genetics Staff

In the title, the prefacing “The” should not be included. The correct title is: Musashi 1 Controls the Splicing of Photoreceptor-Specific Exons in the Vertebrate Retina. The correct citation is: Murphy D, Cieply B, Carstens R, Ramamurthy V, Stoilov P (2016) Musashi 1 Controls the Splicing of Photoreceptor-Specific Exons in the Vertebrate Retina. PLoS Genet 12(8): e1006256. doi:10.1371/journal.pgen.1006256

The publisher apologizes for the error.
