# Supplementary figures and images for: The Musashi 1 Controls the Splicing of Photoreceptor-Specific Exons in the Vertebrate Retina
Source: PLoS Genet. 2016 Aug 19;12(8):e1006256. doi: 10.1371/journal.pgen.1006256 (PMC4991804; doi:10.1371/journal.pgen.1006256)

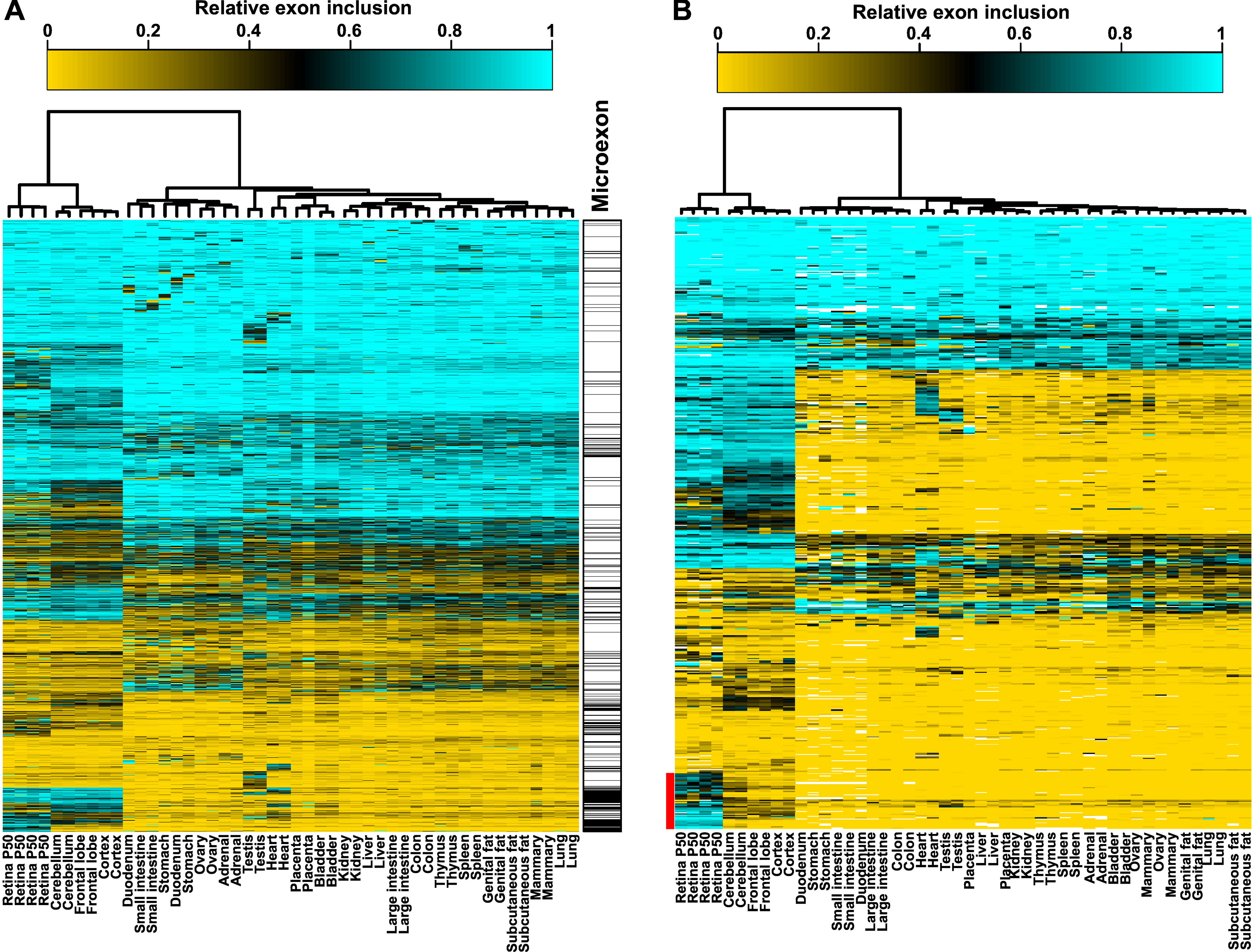

Supplement: S1 Fig — (A) Heat map showing unsupervised hierarchical clustering of a panel of mouse tissues based on the inclusion levels of 8539 alternative exons. Microexons of 30nt or less in length are annotated on the right. Retinal samples form an independent cluster which is related to the cluster formed by the samples from the central nervous system and show frequent use of microexons. Exon in transcripts that are not expressed in the majority of the samples are not included in the heatmap. Missing data points are in white color. (B) Unsupervised hierarchical clustering of tissue samples based on the inclusion levels of 483 microexons shows elevated microexon use in neuronal tissues. A subset of the microexons marked with a red box on the left of the heat map are specifically included in retinal transcripts. (TIF) [file pgen.1006256.s001.tif]

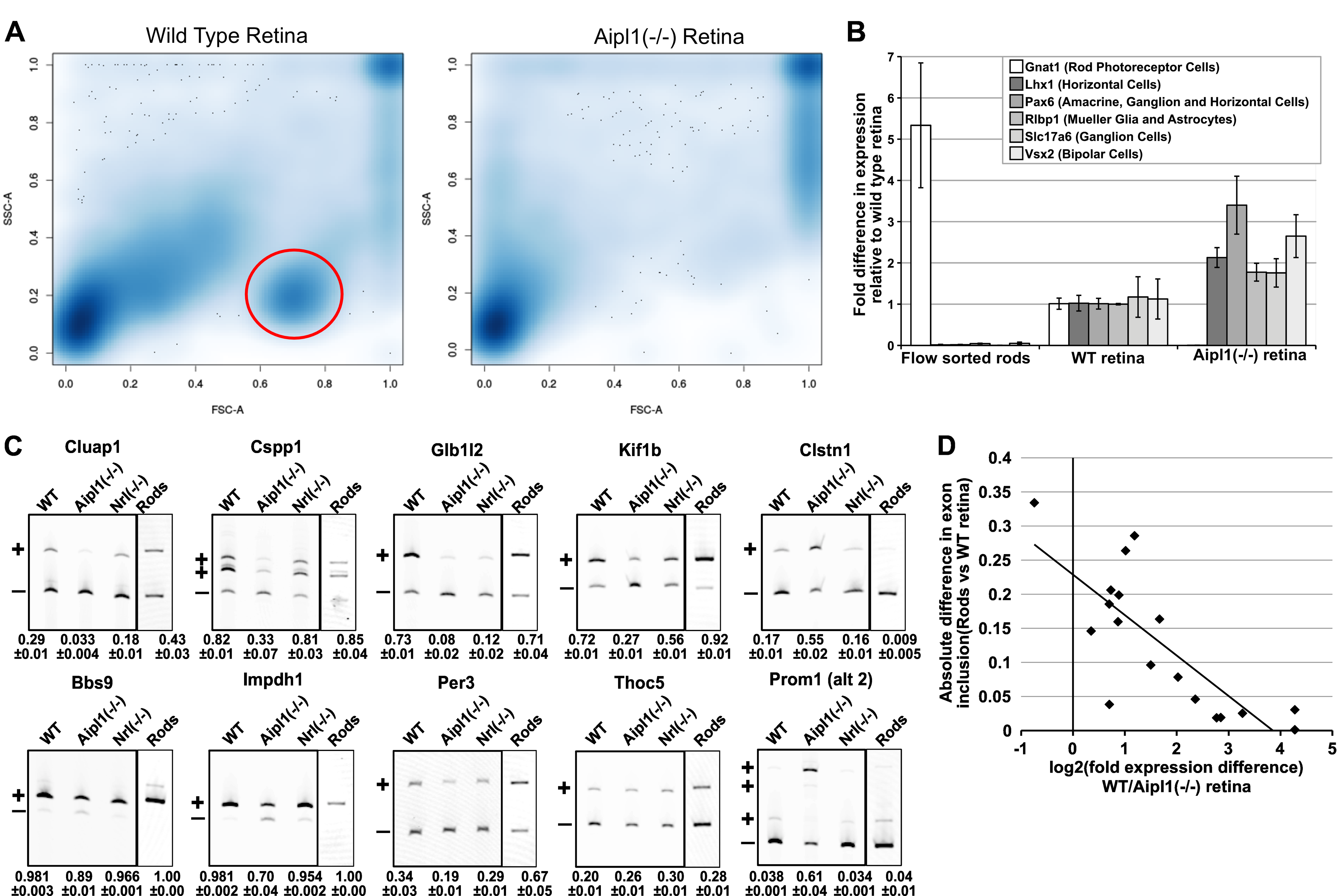

Supplement: S2 Fig — (A) Forward (FSC) vs Side scatter (SSC) scatter plots of dissociated retina from wild type and Aipl1(-/-) mice. The rod photoreceptor population is circled in red on the plot of the wild type retina cells. The rod population is absent from the Aipl1 knockout retina. (B) Quantitative RT-PCR analysis of the expression of photoreceptor, inner neuron and glial markers in the flow sorted rod photoreceptor cells, wild type retina and retina from Aipl1(-/-) mice. The expression levels of all marker genes are normalized to the levels in wild type retina. High levels of the rod transducin (Gnat1) are readily detectable in the flow sorted rod cell population, while the levels of inner neuron and glial cell markers were at or below the assay detection limits. (C) RT-PCR analysis of alternative splicing in wild type retina, Aipl1(-/-) retina, Nrl(-/-) retina and flow sorted rod photoreceptors. The bands corresponding to the exon skipped and exon included mRNA isoforms are labeled with ‘+’ and ‘-’, respectively. The relative exon inclusion and standard error of three independent replicates are shown below each lane. (D) The difference in exon inclusion between rod photoreceptor cells and whole retina inversely correlates with the expression level of the gene in photoreceptors, approximated by the fold change in the expression between wild type and Aipl1 retina. The exon inclusion and gene expression levels were determined by RT-PCR and RNA-Seq, respectively. The inverse correlation is due to the mixed cell type composition of the whole retina and illustrates a limitation in our approach that may prevent the reliable discovery of photoreceptor specific splicing variants of genes with relatively low expression levels in photoreceptors compared to inner neurons. (TIF) [file pgen.1006256.s002.tif]

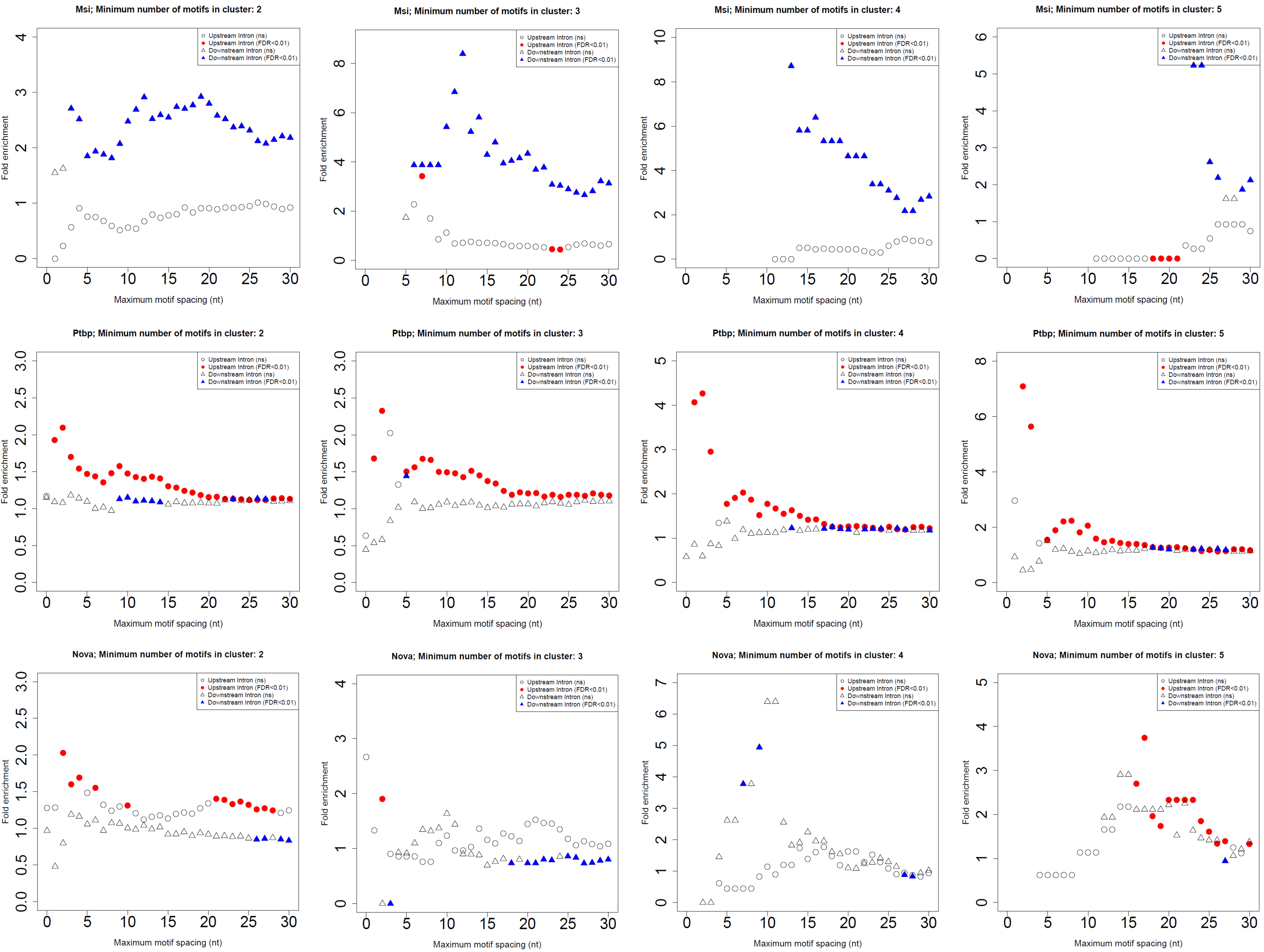

Supplement: S3 Fig — Clusters with minimum size of 2, 3, 4 or 5 motifs were tested for each protein. The spacing between the motifs in a cluster was varied from 0 to 30nt (X—axis). Enrichment upstream or downstream of the exons is plotted with circles and triangles, respectively. Statistically enriched clusters are represented by filled markers using red or blue colors for positions upstream or downstream of the exon, respectively. (TIF) [file pgen.1006256.s003.tif]

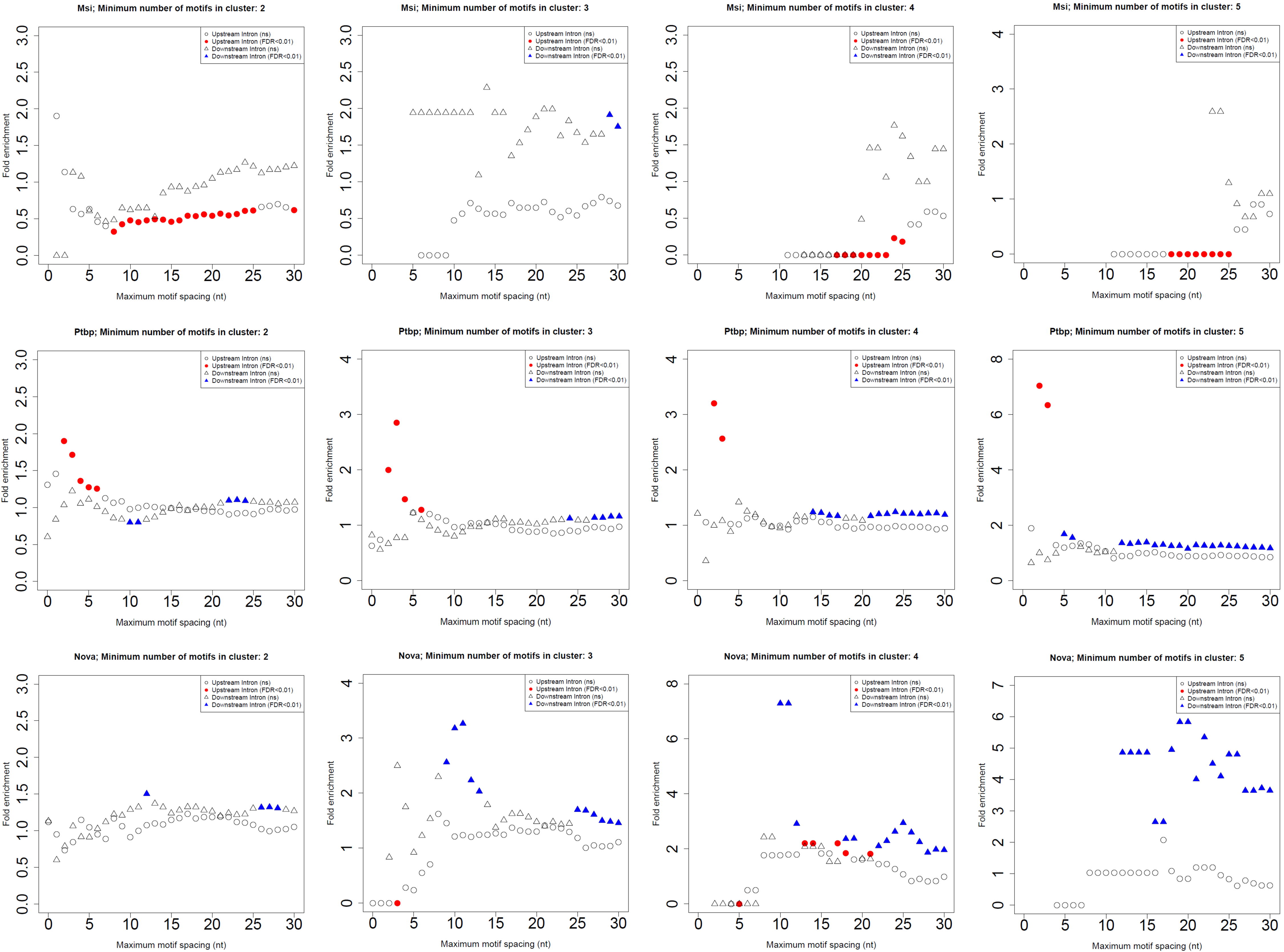

Supplement: S4 Fig — Clusters with minimum size of 2, 3, 4 or 5 motifs were tested for each protein. The spacing between the motifs in a cluster was varied from 0 to 30nt (X—axis). Enrichment upstream or downstream of the exons is plotted with circles and triangles, respectively. Statistically enriched clusters are represented by filled markers using red or blue colors for positions upstream or downstream of the exon, respectively. (TIF) [file pgen.1006256.s004.tif]

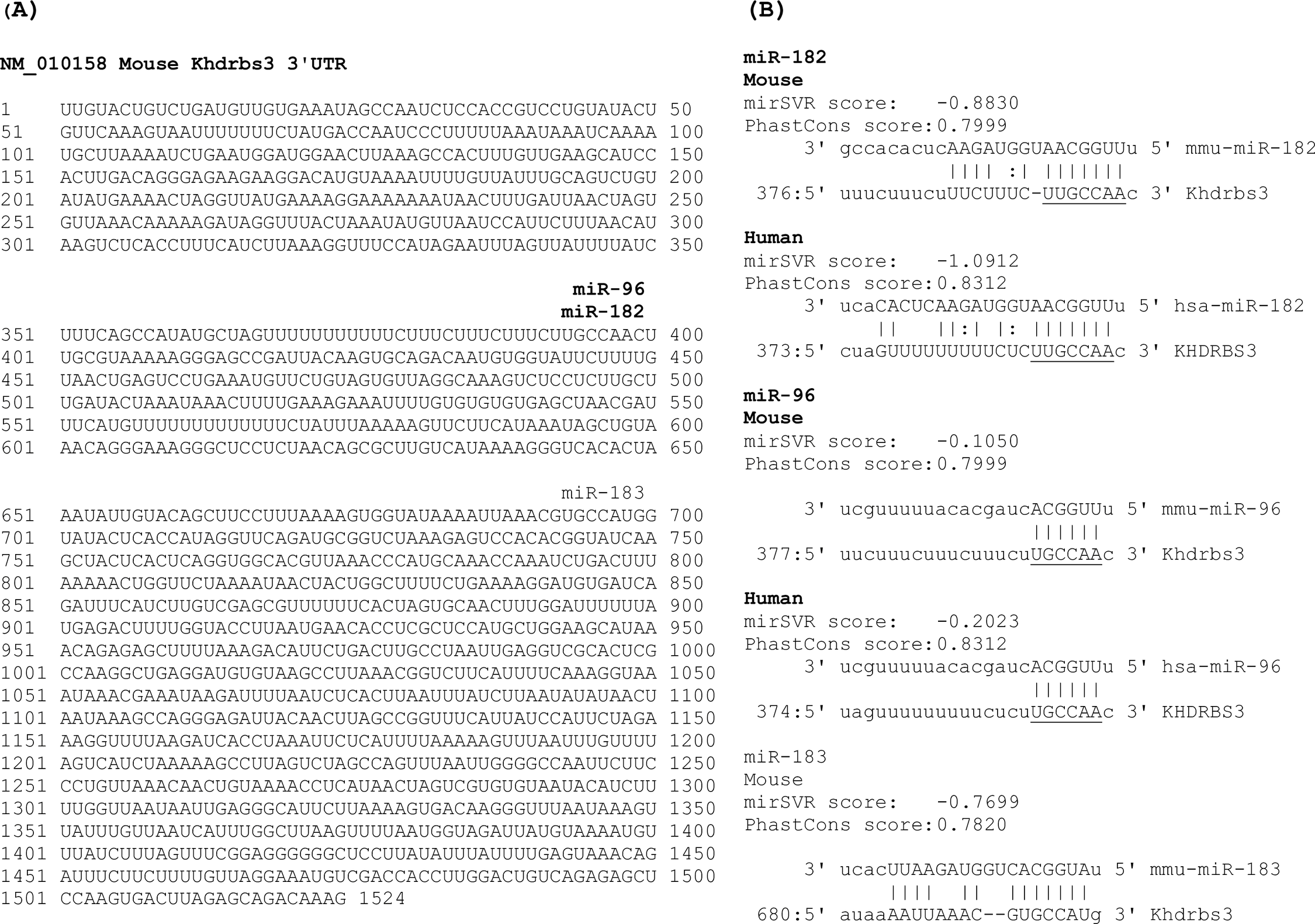

Supplement: S5 Fig — (A) Predicted binding sites for retinal micro-RNAs in the 3'-UTR of Khdrb3. Binding sites conserved between mouse and human are shown in bold typeface. (B) Alignment of the retina specific micro-RNAs to the predicted binding sites. Seed sequences conserved between mouse and human are underlined. Each alignment is accompanied with mirSVR score representing the predicted efficiency of the target site (lower score means higher efficiency) and PhastCons sequence conservation score [76]. (TIF) [file pgen.1006256.s005.tif]

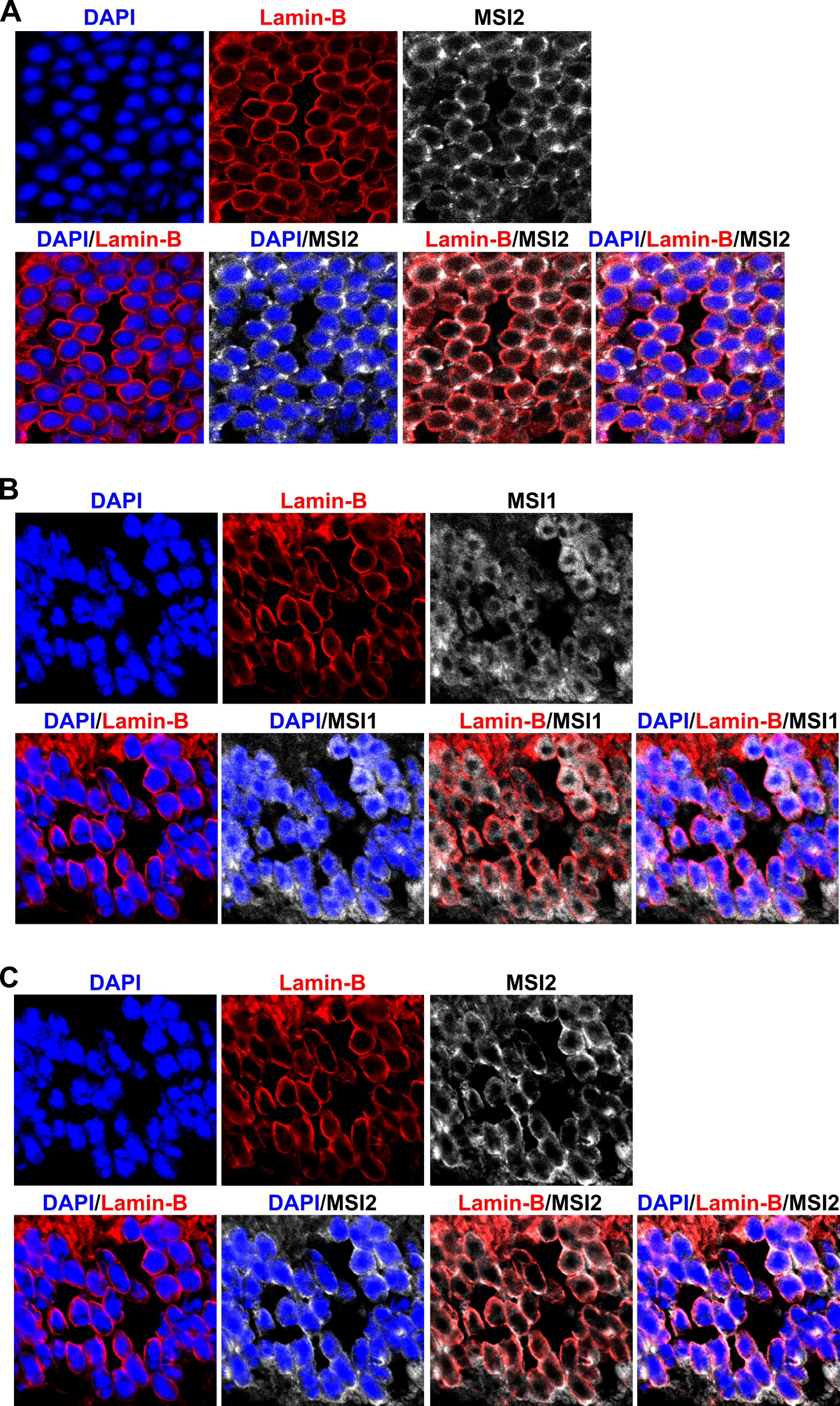

Supplement: S6 Fig — (A) Immunofluorescence detection of MSI2 in the outer nuclear layer of wild type mouse retina (6μm sections). The nuclear envelope is stained with Lamin-B antibody (red). MSI2 staining is shown in gray. The nuclear DNA is stained with DAPI (blue). (B and C) Immunofluorescence detection of MSI1 and MSI2, respectively, in the outer nuclear layer of NRL(-/-) mouse retina (6μm sections). The nuclear envelope is stained with Lamin-B antibody (red). Musashi protein staining is shown in gray. The nuclear DNA is stained with DAPI (blue). The Musashi proteins were visualized on the same section using rat -anti-MSI1 and rabbit anti-MSI2 antibodies in combination with anti-rat AF647 and anti-rabbit AF655 secondary antibodies. (TIF) [file pgen.1006256.s006.tif]

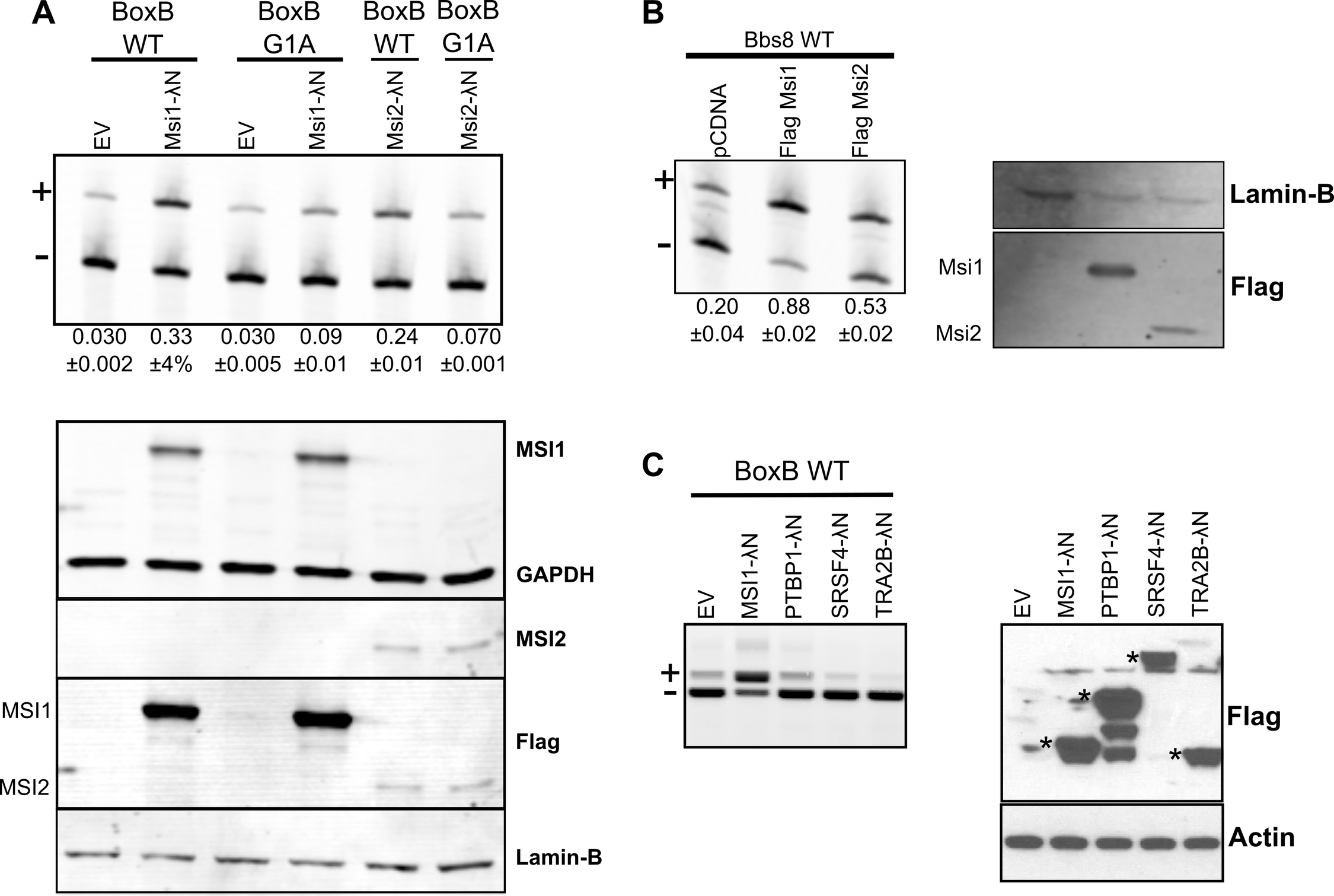

Supplement: S7 Fig — (A) RT-PCR analysis of the splicing of the wild type and G1A minigenes after co-transfection with empty vector or vectors expressing MSI1- λN and MSI2- λN fusions (top). The exon included and exon skipped isoforms are indicated with ‘+’ and ‘-’, respectively. Relative exon inclusion levels with standard error are shown below each lane. Below, western blot shows the expression levels of the MSI1- λN and MSI2- λN proteins. GAPDH and Lamin-B are used as loading controls. (B) RT-PCR analysis of the wild type and mutant Ttc8 exon 2A minigene transcripts after co-transfection with construct expressing flag-tagged MSI1 and MSI2 proteins. The exon included and exon skipped isoforms are indicated with ‘+’ and ‘-’, respectively. Relative exon inclusion levels with standard error are shown below each lane. Below, western blot shows the expression levels of the MSI1 and MSI2 proteins. Lamin-B is used as loading control. (C) RT-PCR (agarose gel on the left) shows that the lambda-N fusion of MSI1 but not PTBP1, SRSF4 and TRA2B, is capable of increasing the inclusion of the test exon when tethered downstream of it. Western blot (right), shows abundant expression of all four fusions. The expected band for each fusion is indicated by an asterisk. (TIF) [file pgen.1006256.s007.tif]

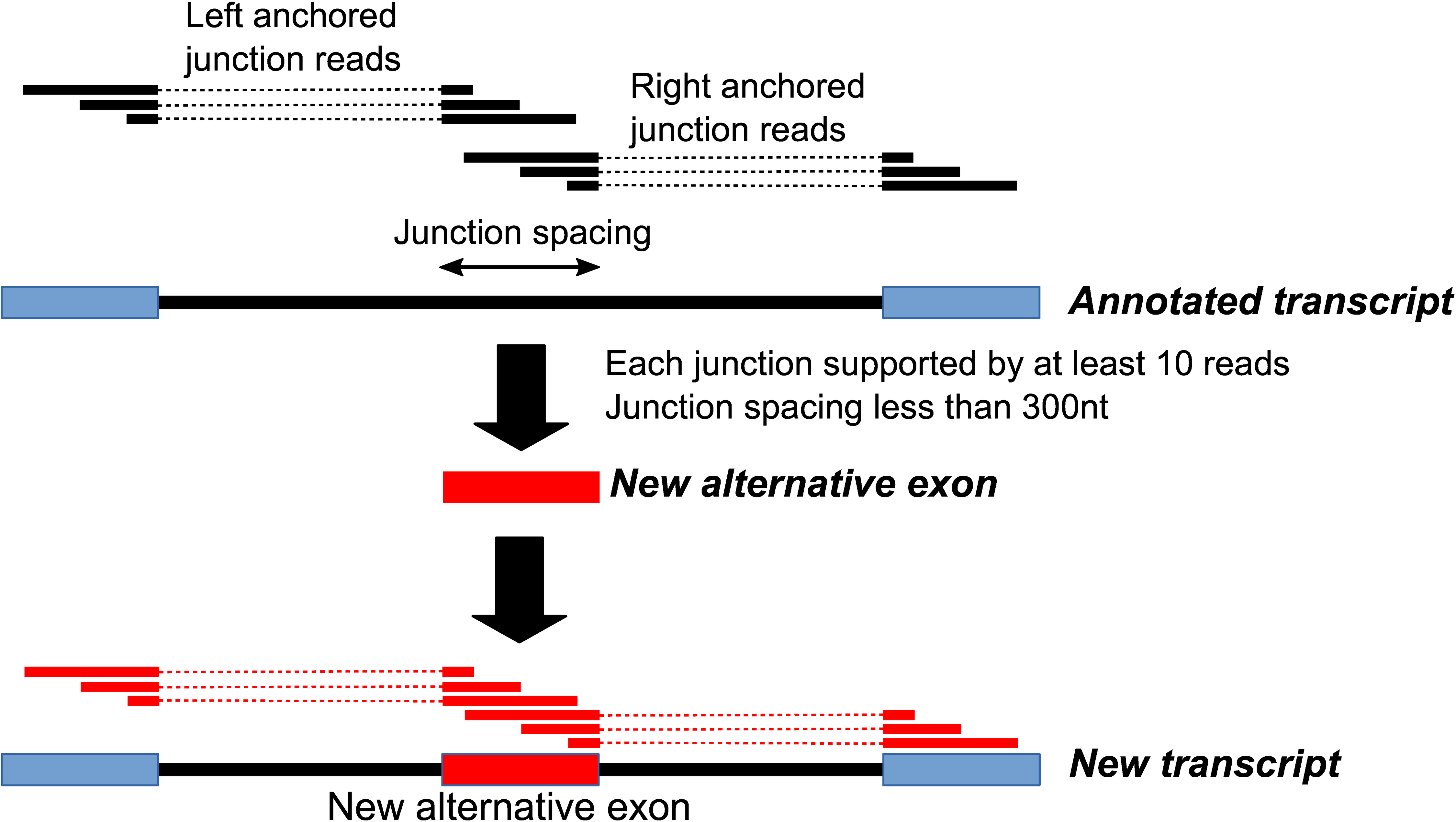

Supplement: S8 Fig — Junction reads that map on one end of an exon in annotated transcripts are used to identify novel exons. A novel exon is defined by two sets of junction reads of at least 10 reads per set, one anchored on the left and a second one anchored on the right to a known exon, that map within a predefined distance (300nt) from each other. (TIF) [file pgen.1006256.s008.tif]
